# Obesity shows preserved plasma proteome in large independent clinical cohorts

**DOI:** 10.1038/s41598-018-35321-7

**Published:** 2018-11-19

**Authors:** Ornella Cominetti, Antonio Núñez Galindo, John Corthésy, Armand Valsesia, Irina Irincheeva, Martin Kussmann, Wim H. M. Saris, Arne Astrup, Ruth McPherson, Mary-Ellen Harper, Robert Dent, Jörg Hager, Loïc Dayon

**Affiliations:** 1 Proteomics, Nestlé Institute of Health Sciences, Lausanne, Switzerland; 2 Nutrition Analytics, Nestlé Institute of Health Sciences, Lausanne, Switzerland; 3 Nutrition and Metabolic Health, Nestlé Institute of Health Sciences, Lausanne, Switzerland; 4 NUTRIM, School for Nutrition, Toxicology and Metabolism, Department of Human Biology, Maastricht University Medical Centre, Maastricht, The Netherlands; 5 Department of Nutrition, Exercise and Sports, Faculty of Science, University of Copenhagen, Copenhagen, Denmark; 6 Ruddy Canadian Cardiovascular Genetics Centre, University of Ottawa Heart Institute, Ottawa, Canada; 7 Department of Biochemistry, Microbiology and Immunology, Faculty of Medicine, University of Ottawa, Ottawa, Canada; 8 Ottawa Hospital Weight Management Clinic, The Ottawa Hospital, Ottawa, Canada; 9 Present Address: Clinical Trial Unit, University of Bern, Bern, Switzerland; 10 Present Address: The Liggins Institute, University of Auckland, Auckland, New Zealand

## Abstract

Holistic human proteome maps are expected to complement comprehensive profile assessment of health and disease phenotypes. However, methodologies to analyze proteomes in human tissue or body fluid samples at relevant scale and performance are still limited in clinical research. Their deployment and demonstration in large enough human populations are even sparser. In the present study, we have characterized and compared the plasma proteomes of two large independent cohorts of obese and overweight individuals using shotgun mass spectrometry (MS)-based proteomics. Herein, we showed, in both populations from different continents of about 500 individuals each, the concordance of plasma protein MS measurements in terms of variability, gender-specificity, and age-relationship. Additionally, we replicated several known and new associations between proteins, clinical and molecular variables, such as insulin and glucose concentrations. In conclusion, our MS-based analyses of plasma samples from independent human cohorts proved the practical feasibility and efficiency of a large and unified discovery/replication approach in proteomics, which was also recently coined “rectangular” design.

## Introduction

In the very recent years, technology improvements have induced an important paradigm shift for biomarker discovery in plasma (as well as in other body fluids and tissues) using mass spectrometry (MS)-based proteomics. Moving from a “triangular” to a “rectangular” approach^1^, we and others have been innovating the clinical proteomic discovery study design from limited sample sizes to much larger cohorts of individuals^2–5^. Common proteomic strategies have applied a “triangular” or funnel design where initial discoveries are obtained on few samples (typically tens), followed by replication and validation of biomarker candidates on increasing numbers of samples (from typically hundreds to thousands). While technical constraints in sample preparation, liquid chromatography (LC) tandem MS (MS/MS) analysis, and bioinformatics have shaped such strategies, new developments in all those areas allow today deeper proteome coverage with higher throughput, offering new possibilities to design proteomic studies. Larger cohorts of human biospecimens (from typically hundreds to thousands) can now be studied with measurements of several hundreds to a thousand of proteins in plasma for instance. In a review, Geyer *et al*. recently used the “rectangular” adjective to conceptualize a clinical study design where both proteomic discovery and validation are performed similarly and on the same large enough number of samples and protein analytes^1^. Practical demonstration of such a concept is however still lacking. Here, we present one of the first examples of such analyses of two large independent cohorts using MS-based proteomics.

Importantly, sample size has been often overlooked in proteomic studies. Several groups have argued that such a shortcut represents a serious caveat that has mostly dampened the proteomic field to translate protein biomarker discoveries into tools of clinical utility^6,7^. In order not to lag behind expectations on the rate of transfer of candidates into the clinics, there is a need to generate knowledge and (candidate) markers that have a better chance to be confirmed afterwards. We have argued that the study of larger cohorts of individuals with proteomics from the initial discovery phase is expected to provide the required statistical power to achieve such goals^3^. In view of the inter-individual variability, typically hundreds to thousands of samples need to be analyzed, resulting from hundreds of subjects at different time points. With our two large proteomic datasets, we further emphasize in this study the relevance of sample size.

To the best of our knowledge, we have gathered the largest combined discovery/validation plasma proteomic studies performed so far with untargeted MS. We have measured and report on two independent cohorts cumulating more than a thousand obese or overweight individuals who followed caloric restriction programs to lose weight; it represents a total of nearly 1500 plasma samples analyzed. In the current work, we provide about 500 unreported proteome profiles of plasma samples collected from obese individuals in clinical practice. As a result, we are able to assess the practical feasibility and efficiency of a “rectangular” design for clinical proteomic research, with emphasis on sample size relevance.

## Results

### Study design and proteomic workflow

**Figure 1:**
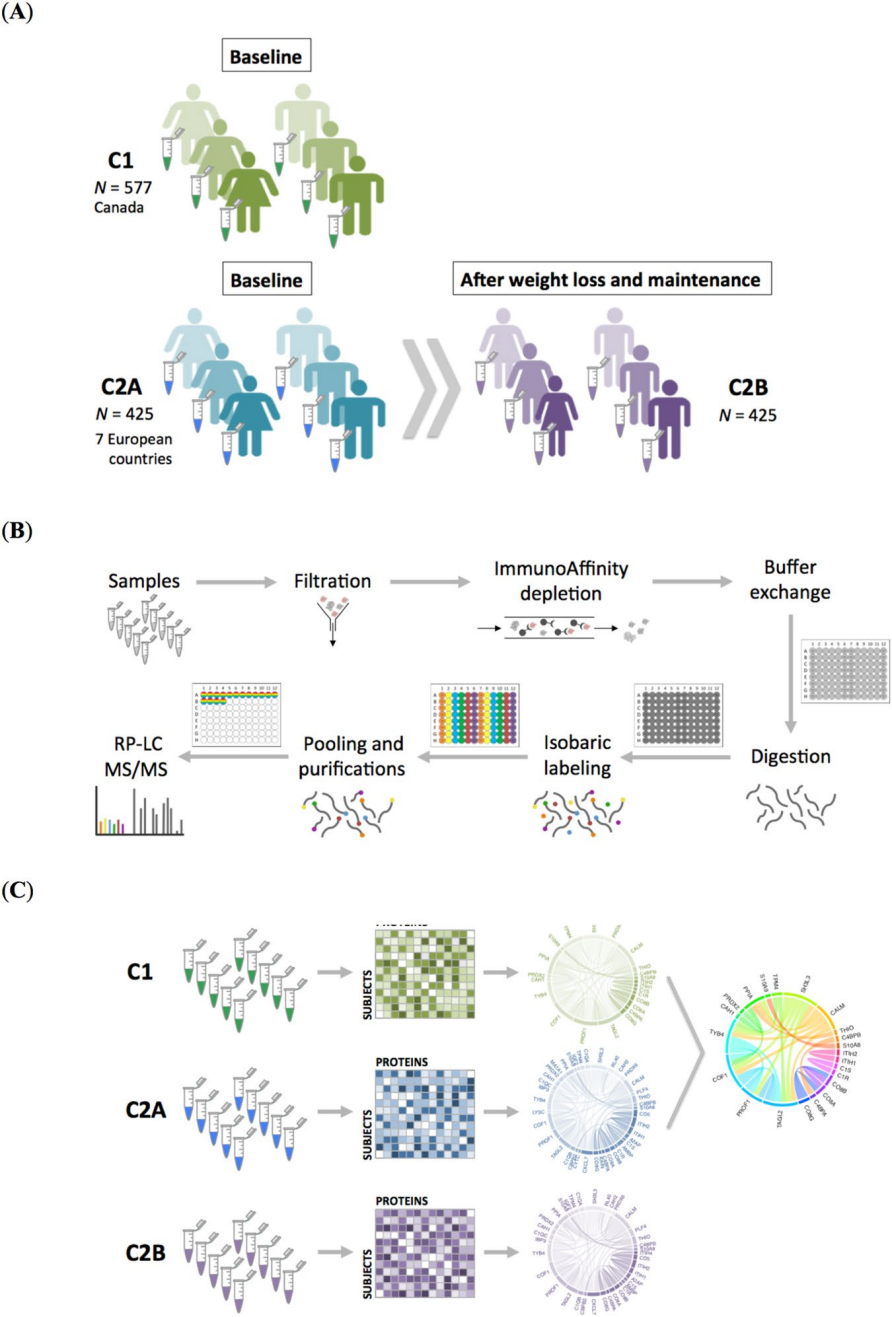
Schematic diagram of (**A**) obese/overweight cohorts’ characteristics, (**B**) experimental workflow and (**C**) study design encompassing the analysis of nearly 1500 individual plasma samples. C1 is a cohort of 577 overweight and obese patients of the Weight Management Clinical program of The Ottawa Hospital. C2 represents a cohort of 425 overweight and obese participants of the European study DiOGenes measured at baseline and at the end of a dietary intervention. Proteomic analysis was performed as previously described (see Experimental Procedures), combining immunodepletion, protein digestion, peptide isobaric labeling (with sixplex technology), and LC-MS/MS analysis. In particular, the last four steps of the sample preparation in (**B**) were performed in an automatic robotic platform as previously described (see Experimental Procedures).

We performed proteomic profiling of nearly 1500 plasma samples belonging to almost one thousand obese and overweight subjects from different countries in the world (Fig. 1A; demographics of the cohorts is presented in Table 1). For that, we used a highly automated shotgun MS-based proteomic workflow relying on isobaric labeling quantification^8^ (Fig. 1B). The first cohort under study was composed of 577 overweight and obese patients of the Weight Management Clinical program of The Ottawa Hospital; those patients underwent low calorie diet (LCD) to reduce their weight during a period of 6 to 12 weeks (see Experimental Procedures for a detailed description). Throughout this manuscript, we refer to cohort C1 to designate this cohort from Canada with plasma samples available at baseline (*i.e*., before intervention; Fig. 1A). The second investigated cohort pertained to the European DiOGenes study^9^. It was composed of 425 overweight and obese participants who followed 8 weeks of controlled LCD, then randomized into 6 months of weight maintenance phase. We refer to the DiOGenes cohort as C2A at baseline and as C2B after both weight loss and maintenance (Fig. 1A). Plasma samples were available at both time points and proteomic profiles had been previously acquired for differential analysis during the DiOGenes intervention^5^. The main purpose of the present work aimed at comparing the plasma proteome profiles of C1 (data never presented before) and C2A (*i.e*., at baseline), representative of overweight and obese phenotypes (Fig. 1C). Demographics and clinical characteristics of the cohorts are detailed in Table 1. C1 is composed of more obese subjects than C2. As expected then, fasting insulin levels are higher in C1 than in C2 and they are higher in males *versus* females for both cohorts.

**Table 1:** Demographics and clinical characteristics of the Canadian C1 and European C2 cohorts.

Cohort	C1		C2	
Variable	Females	Males	Females	Males
N	403	174	273	152
Age [years]	48 ± 12	50 ±11	42 ± 6	43 ± 6
BMI at baseline [kg/m^2^]	44.80 ± 7.44	44.94 ± 7.36	33.88 ± 4.70	33.83 ± 4.49
BMI after intervention [kg/m^2^]	40.93 ± 6.90	40.29 ± 6.45	30.48 ± 4.39	30.44 ± 4.21
WC at baseline [cm]	163.53 ± 6.58	177.82 ± 12.96	103.59 ± 11.72	113.06 ± 11.61
WC after intervention [cm]	115.41 ± 13.14	128.56 ± 14.76	94.16 ± 11.25	101.15 ± 11.24
Fasting insulin [mIU/L derived]	18.72 ± 17.74	26.34 ± 20.81	10.90 ± 10.90	13.46 ± 11.99
Fasting glucose [mmol/L derived]	5.87 ± 2.16	6.92 ± 2.77	5.03 ± 0.63	5.25 ± 0.48
Cholesterol, fasting [mmol/L]	4.736 ± 0.98	4.07 ± 1.00	4.73 ± 0.97	4.97 ± 1.12
HDL, fasting [mmol/L]	1.21 ± 0.29	0.97 ± 0.22	1.30 ± 0.33	1.04 ± 0.24
LDL, fasting [mmol/L]	2.77 ± 0.87	2.29± 0.90	2.90 ± 0.85	3.18 ± 0.97
Triglycerides, fasting [mmol/L]	1.72 ± 0.97	1.92 ± 1.08	1.21 ± 0.56	1.64 ± 0.72

Data are presented as mean ± standard deviation (SD); N: Number of individuals; BMI: Body mass index; WC: Waist circumference; HDL: High-density lipoprotein; LDL: Low-density lipoprotein.

**Figure 2:**
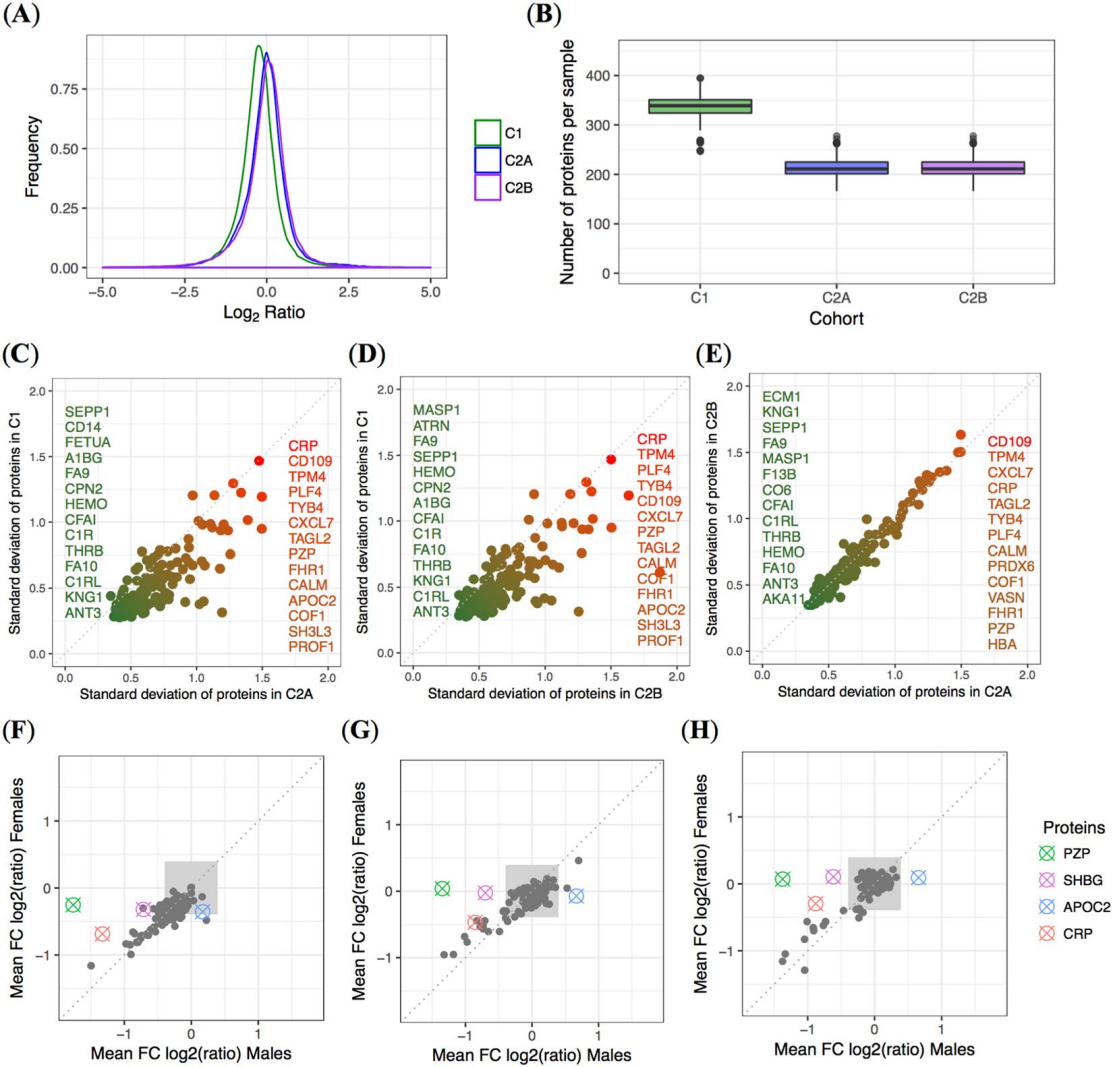
(**A**) Distributions of Log2 of the protein ratio fold-changes with respect to pool samples and (**B**) number of proteins identified per sample for the three cohorts (*i.e*., C1, C2A, and C2B). Scatter plots for individual protein SD obtained for the C1 cohort *versus* the C2 cohorts, (**C**) C2A and (**D**) C2B, and between the two collection days in C2 (**E**). The least variable - on average between the measurements - (green color) and most variable (red to orange color) commonly quantified proteins between the cohorts are displayed. Gender differences in the (**F**) C1 and (**G**) C2A, and (**H**) C2B. Mean relative quantitative proteins values, measured as the Log2 of the protein ratio fold-changes with respect to pool samples. The gray square represents the area for values comprised within the estimated technical variance. Only proteins with a high mean fold change difference between genders are shown in color. The full list of significant gender-specific proteins, are presented in Table 2. For C2, data were averaged over seven European collection centers.

Following the proteomic workflow depicted in Fig. 1B that relies on isobaric tagging for sample multiplexing, LC-MS/MS was performed in duplicate for a total of 843 LC-MS/MS analyses (raw data are available at ProteomeXchange; see Experimental Procedures). We identified in total 507 proteins in C1 and 364 proteins in C2A and C2B. Of those, 299 and 182 proteins were constantly quantified (*i.e*., proteins with less than 30% missing quantitative data points) in C1 and in C2A/B, respectively. It resulted in an overlap between the cohorts of 179 quantified proteins for comparison. The baseline datasets (*i.e*., C1 and C2A) achieved a data completeness of 97.38% for 179 proteins measured in 1002 individuals. The quantitative data exhibited an overall normal distribution (Fig. 2A).

The number of identified proteins *per* sample was on average 337 (±21) for C1 and 213 (±19) for C2A/B in duplicate LC-MS/MS analyses (Fig. 2B; samples of C2A and C2B were analyzed together with LC-MS/MS after sixplex isobaric labeling and sample pooling as previously reported)^5^. Plasma sample collection, quality, and integrity in each cohort may have influenced this proteome coverage difference, as the exact same proteomic workflow was applied for C1, measured here, and C2A/B, measured previously. Quantitative precision of a spiked internal standard (*i.e*., bovine β-lactoglobulin (LACB)) in all samples was below 12% in both datasets. Overall, this last metric confirmed the quality of the quantitative data before their further exploration, in one of the largest combined plasma proteomic dataset ever generated using LC-MS/MS (*i.e*., ~ 1560 individual plasma samples together with ~ 880 plasma pools).

### Replication of the variability of plasma proteomes

We assessed the variability of the proteins between both C1 and C2A/B cohorts. We found a very high correlation (Pearson correlation coefficients >0.76) between the measurement variability of the proteins among the cohorts (Fig. 2C–E), despite C1 and C2A/B data belonging to different and heterogeneous populations (see Experimental Procedures). On top, difference in sample collection was present (see also Experimental Procedures) and proteomic analyses were performed independently in two different exercises distant from several months for C1 and C2A/B despite using the same equipment and workflow.

C-reactive protein (UniProtKB entry name: CRP) appeared consistently as one of the plasma proteins with highest variability (Fig. 2C–E). This observation could be because these populations were composed of either obese or overweight individuals with varying degrees of inflammation, often transient, for which CRP is a known marker. While CRP levels are strongly associated with body weight^10^, reduced with weight loss and increased with weight gain, they are also affected by concomitant treatments including statins and nonsteroidal anti-inflammatory drugs. Among others, CRP measured with LC-MS/MS was also previously shown to strongly vary between individuals^4^, as an acute phase marker for inflammation in the body. Some proteins presenting high variability might be more deeply studied as this degree of variance can be attributed to the randomness of the measurements (biological and technical) but also to very different underlying phenotypes in the studied populations (see next section on gender specificity).

Among the least variable proteins between individuals, we found several members of the coagulation and complement cascades. Few of those have been previously shown to be also stable overtime in the plasma proteome^11^. Antithrombin-III (ANT3), kininogen-1 (KNG1), complement C1r subcomponent-like protein (C1RL), coagulation factor X (FA10), prothrombin (THRB), complement factor I (CFAI), hemopexin (HEMO), coagulation factor IX (FA9), and selenoprotein (SEPP1) were among the least variable proteins in all three cohorts (*i.e*., 9/14 proteins, representing 64% of the lists of least variable proteins). This constitutes a very strong result on the capabilities of MS-based proteomic profiling to provide reliable measurements in independent large clinical cohorts. Comparing only C1 and C2A (*i.e*., at baseline), ANT3, KNG1, C1RL, FA10, THRB, complement C1r subcomponent (C1R), CFAI, HEMO, carboxypeptidase N subunit 2 (CPN2), FA9, α-1B-glycoprotein (A1BG), and SEPP1 were the common less variable proteins in plasma samples (12 of the 14 less variable proteins, *i.e*., 86%, in C1 are also among the 14 less variable proteins in C2A).

In general, the standard deviations (SDs) for the commonly measured proteins were smaller in C1 than in C2. This could be explained by the fact that the C1 population was more uniform (see Supplementary Material); on top, there was only one collection center in one country (*i.e*., Ottawa, Canada), unlike C2, with its seven collection centers considered here, each in a different European country (see Experimental Procedures), and with a center effect previously identified^3^.

### Plasma proteome and gender

As shown in our previous work^3^, a few proteins showed differences between females and males in C2A and C2B (Fig. 2G,H). The three gender-specific proteins identified, namely pregnancy zone protein (PZP), sex hormone-binding globulin (SHBG), and apolipoprotein C-II (APOC2) replicated also as gender-specific proteins in the C1 cohort (Fig. 2F). The observed high variability of PZP and APOC2 (Fig. 2C-E) might be therefore mostly driven by the important differences between males and females. We believe assessing those gender proteins can be a valuable quality check of human plasma proteomic data.

CRP (Fig. 2F–H) was measured at higher levels in males than in females in some studies^12–14^. However, those results may be considered as inconclusive, as other studies showed opposite results^15,16^ or no significant difference^17^. As in both C1 and C2A cohorts, men presented higher abdominal fat (Table 1), the observed differences in CRP levels may result from a more profound acute inflammation state in the male than in the female subjects.

It is a known fact that several covariates affect the measurements of proteins in biological fluids such as human plasma. Those confounders should always be considered when designing clinical research studies. We performed a comparative analysis of these possible confounders in our study. Following a similar approach to that of Enroth *et al*.^18^, we displayed significant effects of anthropometric variables on the proteins. The figure and a breakdown of this analysis per cohort is presented in the Supplementary Material (Supp. Figs S1 and S2). Results of statistics are given in Tables 2–4 for gender, age, and body mass index (BMI), respectively.

**Table 2:** Proteins significantly associated with gender for the three cohorts.

Cohort	C1		C2A		C2B	
Protein	Coefficient estimate	Corrected *p*-value	Coefficient estimate	Corrected *p*-value	Coefficient estimate	Corrected *p*-value
PZP	1.480	1.90E-46	1.370	5.07E-24	1.422	5.62E-23
SHBG	0.408	2.87E-07	0.684	1.41E-17	0.734	1.49E-20
APOC2	−0.501	6.42E-05	−0.711	1.38E-10	−0.539	1.29E-06
CRP	0.580	5.23E-05	0.384	3.94E-02	0.591	1.98E-03
A2GL	0.115	1.54E-02	0.286	1.18E-07	0.308	5.81E-09
AFAM	−0.154	2.54E-04	−0.150	1.51E-02	−0.125	4.54E-02
CBG	0.179	2.77E-03	0.175	3.47E-02	0.275	1.61E-03
CERU	0.211	9.55E-08	0.211	6.70E-03	0.358	9.84E-07
FETUB	0.107	3.07E-02	0.190	1.08E-02	0.182	3.21E-02
PHLD	−0.207	2.06E-07	−0.257	3.45E-04	−0.187	2.96E-02
PRAP1	−0.211	2.13E-05	−0.201	8.94E-03	−0.176	4.42E-02

**Table 3:** Proteins significantly associated with age for the three cohorts.

Cohort	C1		C2A		C2B	
Protein	Coefficient estimate	Corrected *p*-value	Coefficient estimate	Corrected *p*-value	Coefficient estimate	Corrected *p*-value
C4BPA	0.010	2.12E-05	0.018	2.63E-02	0.021	6.64E-03
C4BPB	0.013	1.17E-05	0.023	2.47E-02	0.024	2.87E-02
CRAC1	0.013	2.35E-09	0.017	1.51E-02	0.022	3.17E-03
FBLN3	0.011	5.06E-15	0.012	1.51E-02	0.012	1.11E-02
HRG	0.013	1.30E-06	0.023	9.76E-03	0.023	8.17E-03
IC1	0.006	1.26E-03	0.020	1.56E-02	0.027	1.98E-03
LUM	0.014	8.36E-18	0.010	4.61E-02	0.013	2.87E-03
PGRP2	0.004	6.20E-03	0.011	2.94E-02	0.013	8.46E-03
TIMP2	0.007	3.44E-07	0.011	2.41E-02	0.014	1.11E-02

On top of the already identified differential proteins between genders by comparing their average between males *versus* females (*i.e*., PZP, SHBG, APOC2, and CRP), using a linear model approach, we determined additional gender-associated proteins such as leucine-rich alpha-2-glycoprotein (A2GL), afamin (AFAM), corticosteroid-binding globulin (CBG), ceruloplasmin (CERU), fetuin-B (FETUB), phosphatidylinositol-glycan-specific phospholipase D (PHLD), and proline-rich acidic protein 1 (PRAP1) (Table 2). Among them, APOC2, AFAM, PHLD, and PRAP1 showed a negative association with the gender parameter (*i.e*., are higher in males than in females). PZP presented the strongest positive association with the gender parameter (*i.e*., largest estimate in Table 2) and APOC2 the strongest negative association. The higher presence of APOC2 in plasma of males has already been reported^19^. Those proteins should therefore be considered with caution when proposed as candidate biomarkers as they might be strongly influenced by gender. Several of these proteins had already been identified to show gender dimorphism in different cohorts and using different proteomic measurement techniques. Such is the case for SHBG and FETUB being higher in females than in males^20^, PZP^21^ and CBG^22^, also higher in females than in males, and PHLD higher in males than in females^21^.

### Plasma proteome and age

The proteins that are significantly associated with age in the three cohorts were C4b-binding protein alpha chain (C4BPA), C4b-binding protein beta chain (C4BPB), cartilage acidic protein 1 (CRAC1), EGF-containing fibulin-like extracellular matrix protein 1 (FBLN3), histidine-rich glycoprotein (HRG), plasma protease C1 inhibitor (IC1), lumican (LUM), N-acetylmuramoyl-L-alanine amidase (PGRP2), and metalloproteinase inhibitor 2 (TIMP2). All these proteins were positively associated with age (positive values in the estimates of Table 3). The age of the population ranged between 16 and 81 years for C1, and 16 and 63 years for C2A/B. It is reasonable to think that if the C2 cohorts also had subjects in the last two decades of the age range of C1, when important physiological and protein changes may occur, more proteins could have been identified to be commonly influenced by age.

### Plasma proteome and BMI

Among the BMI-protein significant associations in these three obese/overweight datasets, we consistently observed complement factor B (CFAB), complement factor H (CFAH), CFAI, CRP, PRAP1, as well as the calprotectin complex formed by proteins S100-A8 (S10A8) and S100-A9 (S10A9). All these proteins were positively associated with CRP presenting by far the strongest association with BMI (*i.e*., largest estimate in Table 4 in each cohort).

**Table 4:** Proteins significantly associated with BMI for the three cohorts.

Cohort	C1		C2A		C2B	
Protein	Coefficient estimate	Corrected *p*-value	Coefficient estimate	Corrected *p*-value	Coefficient estimate	Corrected *p*-value
CFAB	0.006	3.74E-02	0.017	3.48E-03	0.012	4.04E-02
CFAH	0.006	1.57E-02	0.022	2.20E-03	0.020	2.18E-03
CFAI	0.006	6.67E-03	0.015	6.70E-03	0.012	3.15E-02
CRP	0.059	4.05E-12	0.111	2.57E-11	0.086	1.29E-06
PRAP1	0.007	4.99E-02	0.038	5.50E-08	0.021	1.67E-02
S10A8	0.013	1.55E-02	0.045	1.07E-06	0.024	3.69E-02
S10A9	0.018	5.65E-04	0.044	3.34E-07	0.031	1.23E-03

### Plasma proteome and other clinical variables

We then looked at markers of obesity and associated co-morbidities and directly compared the results of C1 and C2A (*i.e*., baseline measurements). Additional results for the C2B dataset are provided in the Supplementary Material, Supp. Fig. S3. We also investigated whether gender or age influenced these results and verified that there were only minor differences after correcting the protein measurements for age and gender (see the results in Supp. Fig. S4 of the Supplementary Material). As a matter of fact, the association significance was mainly decreased for protein correlations with glucose and HDL levels. We observed good agreements of the clinical variable-protein associations between both C1 and C2A cohorts (Supplementary Material, Supp. Fig. S5). More precisely, we observed an average agreement of 60% between the significantly associated proteins in the cohorts.

**Figure 3:**
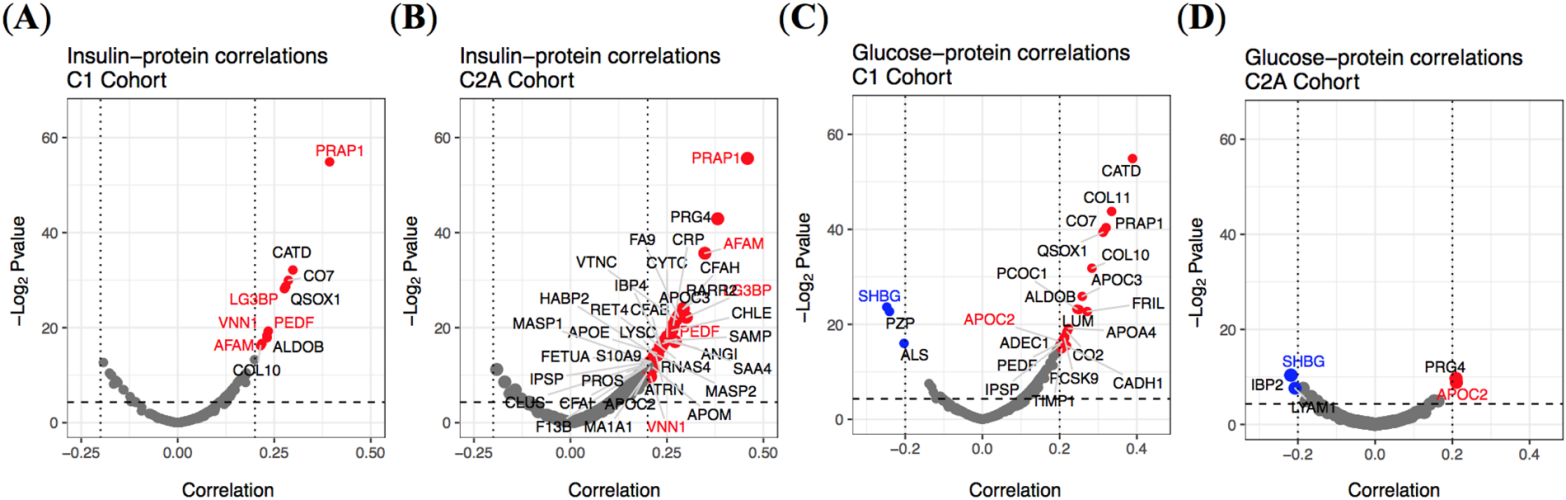
Clinical variable-protein correlations for C1 and C2A, shown as volcano plots. (**A**,**B**) Fasting insulin [mIU/L derived], (**C**,**D**) fasting glucose [mmol/L derived]. The horizontal dashed line in each plot represents the threshold of significance of adjusted *p*-value ≤ 0.05 and the vertical dotted lines represent the limits of −0.2 and 0.2 of the Spearman correlation coefficients. Only the names of the proteins significantly regulated are shown, and they are colored if they are significant for both cohorts. Red corresponds to positive correlated proteins (*i.e*., on the right) and in blue are depicted the negatively correlated proteins (*i.e*., on the left).

In particular, several proteins significantly associated with insulin and glucose levels in blood (Fig. 3):

- ***Fasting insulin****.* PRAP1 was found to strongly correlate (positively) with fasting insulin for both C1 and C2A datasets (Fig. 3A,B), also when accounting for gender and age (Supplementary Material, Supp. Fig. S4C,D). Other proteins, also with positive correlations to fasting insulin for both baseline cohorts were pantetheinase (VNN1), AFAM, pigment epithelium-derived factor (PEDF), and galectin-3-binding protein (LG3BP), although to a lesser degree than PRAP1. Fasting insulin was correlated positively with a large number of proteins in C2A (Fig. 3B).
- ***Fasting glucose****.* High positive correlations and some negative correlations of some proteins were found for C1 data with fasting glucose, specifically cathepsin D (CATD) (Fig. 3C). CATD has been shown to be associated with indices of insulin-resistance homeostasis model assessment (*i.e*., HOMA computed from fasting glucose levels) in newly diagnosed type 2 diabetes (T2D) patients. For C2A (Fig. 3D), the correlations were very small yet significant. SHBG was found in the two cohorts as negatively correlated with fasting glucose while APOC2 was positively correlated with fasting glucose.

On the subsequent analyses, we further focused only on the baseline cohorts (*i.e*., C1 and C2A) in order to identify the commonalities between these two large obese/overweight populations. In the Supplementary Material, Supp. Figs S6 and S7, complementary results for the C2B dataset after the dietary intervention are provided.

### Plasma proteins and their association maps in obesity and overweight

**Figure 4:**
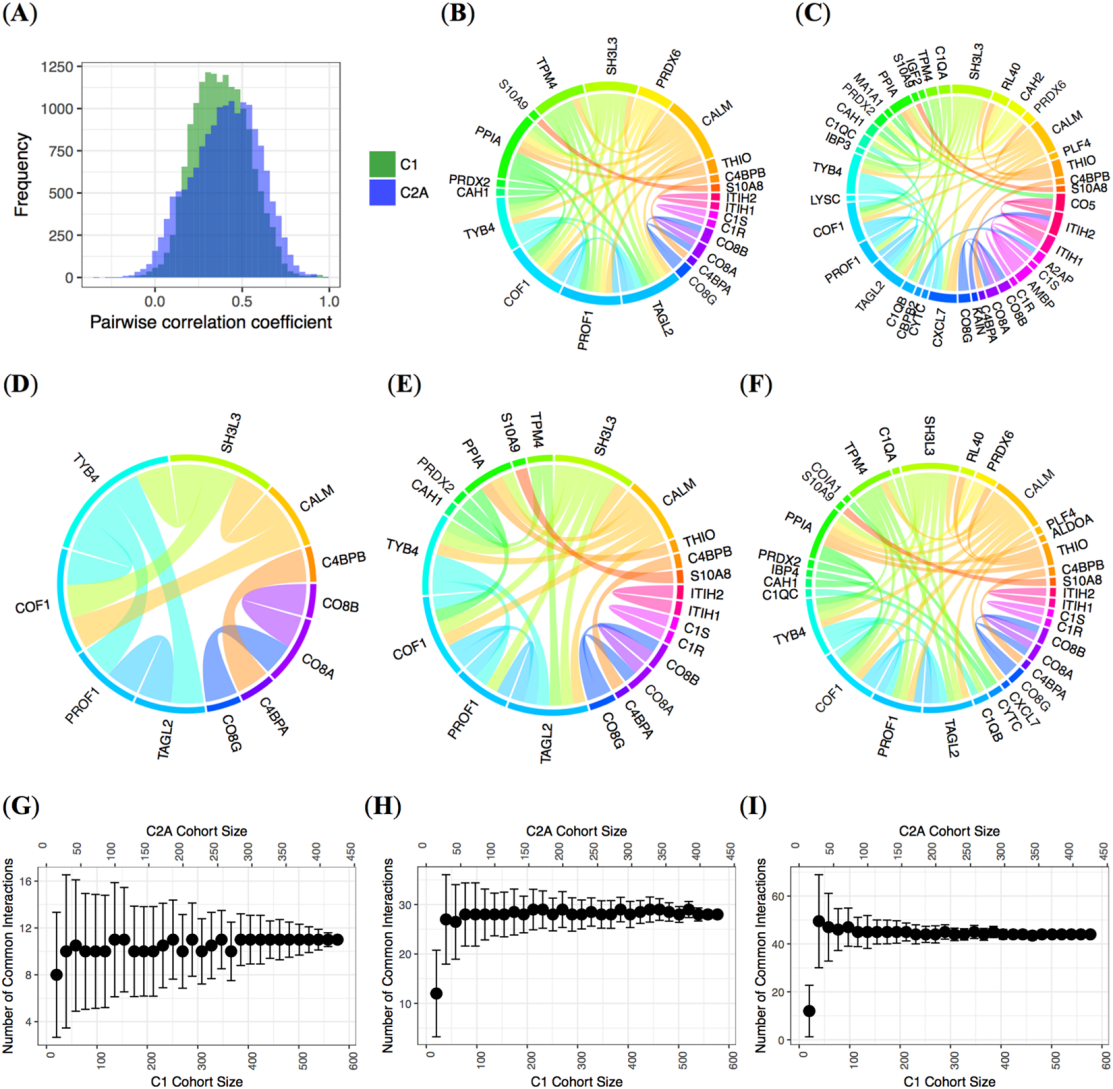
(**A**) Histogram of pairwise correlation coefficients for C1 (in green) and C2A (in blue). Chord diagrams representing significant correlations between pairs of proteins in (**B**) C1 (Spearman, *R* >  0.85) and (**C**) C2A (Spearman *R* > 0.85). Levels of significance were corrected for multiple testing using the Benjamini-Hochberg procedure. Chord diagrams representing significant common inter-protein relationships between baseline cohorts, C1 and C2A for decreasing stringency: thresholds for Spearman correlation coefficients of (**D**) 0.90, (**E**) 0.85, and (**F**) 0.80. Number of significant pairs was (**D**) 11, (**E**) 28, and (**F**) 44, respectively. Levels of significance were corrected for multiple testing using the Benjamini-Hochberg procedure. Number of common significant interactions between the C1 and C2A cohorts after randomly removing a given percentage of their total number of subjects for decreasing stringency; thresholds for Spearman correlation coefficients of (**G**) 0.90, (**H**) 0.85 and (**I**) 0.80 were used. The dots represent the median value of the number of interactions identified among all the iterations, and the bars represent the corresponding SDs. The top *x*-axis corresponds to the size of the C2A cohort, while the bottom *x*-axis represents the number of subjects in the C1 cohort.

With our large and unique MS-based proteomic datasets, we then deciphered an interaction map of the plasma proteins in obese/overweight individuals. Large numbers of pairs or groups of plasma proteins showed high correlation between them, given their participation in same complexes, or the same signaling pathways, biological processes or other types of interactions. Studying the frequency of correlation coefficients in the datasets, we observed that the distributions differed between the C1 and C2A cohorts (Fig. 4A).

With chord diagrams, we represented the significant correlations between any pair of proteins in both C1 and C2A (Fig. 4B,C). Results for these analyses including C2B are shown in Supp. Figs S6 and S7. Inter-protein correlations in C1 (Fig. 4B) were for the vast majority smaller than in C2A (Fig. 4C). As expected, we observed strong repeated associations between protein components of same complexes (*e.g*., calprotectin composed of S10A8 and S10A9; membrane attack complex composed of complement component C8 alpha chain (CO8A), complement component C8 beta chain (CO8B) and complement component C8 gamma chain (CO8G); and C4BPA composed of 7 identical C4BPA chains and one unique C4BPB chain), members of the complement system, or proteins from the same families (*e.g*., inter-alpha-trypsin inhibitor heavy chains (ITIHs)).

All the strongest correlations were positive. The largest negative correlations were below 0.40, while the highest positive correlation were above 0.90. This was likely the case because strong positive correlations may be due to strong physical binding of proteins, while anti-correlations between proteins might be caused by subtle regulatory relationships for instance.

In Fig. 4D–F, the chord diagrams of the common interactions found in both C1 and C2A are represented, highlighting the plasma protein-protein relationships at different levels of stringency. The proteins of the complement system were here again consistently highly correlated with each other in plasma. Interestingly, another set of proteins composed of calmodulin (CALM), SH3 domain-binding glutamic acid-rich-like protein 3 (SH3L3), thymosin beta-4 (TYB4), cofilin-1 (COF1), profilin-1 (PROF1), and transgelin-2 (TAGL2) also clearly emerged (Fig. 4D). Those interactions suggest that some protein measurements might be used as proxy of other proteins in plasma. Applying less stringency (Fig. 4E,F), some other specific associations appeared, somehow independent of the previously mentioned (*e.g*., S10A8 and S10A9, complement C1s subcomponent (C1S) and C1R, and ITIH1 and ITIH2).

### Relevance of sample size in MS-based proteomic profiling of human plasma

In order to observe how the sample size of our cohorts contributed to the number and validity of associations revealed, we repeated the previous protein-protein correlation analysis in smaller subsets of these cohorts. For this, randomly, we removed an increasing percentage of the subjects from the two cohorts C1 and C2A and observed the average results of the number of associations identified. To derive a robust estimate of the number of correlations among the proteins, we performed bootstrap and repeated 100 times this approach^23,24^. As expected, for increasingly large sample sizes, the average stabilizes and the range becomes narrower (law of large numbers)^25^.

In Fig. 4G–I, for small datasets (less than 25 subjects considered) the number of common significant interactions (adjusted *p*-value ≤ 0.05) was roughly one third of the ones found for larger sample sizes, exemplifying that many plasma discovery proteomic results reported so far are underpowered indeed. In our study, the variability on the number of identified interactions decreased when the cohort size increased. For example, in our data, to obtain SD smaller than 10% on the number of common interactions, over 100 subjects were needed. The relative variability decreased as the stringency threshold decreased (from Fig. 4G to Fig. 4I). Therefore, to obtain high confidence in the deciphered protein-protein interactions, it is mandatory to deal with sufficiently large cohorts. The averaged 11 common interactions for the first point on the left in Fig. 4H,I (which were obtained from 100 iterations and therefore with more than the actual small numbers of samples indicated in the figures), corresponded to the interactions observed for the most stringent of the thresholds, *i.e*., the one shown in the last point on the right of Fig. 4G. These proteins were so strongly correlated, that even for some small subsets of the cohorts, they could potentially be identified (but with the risk of finding together false positive and negative discoveries as illustrated by the large SD bars obtained with such small numbers of samples).

## Discussions

In this work, we demonstrated that MS is a robust technology for protein biomarker discovery and replication in human blood. MS-based proteomic workflows can be used in clinical research studies of large cohorts. In particular, shotgun proteomics using relative quantification techniques can generate highly consistent and reproducible results. It can provide new insights into complex phenotypes^4,5^ amenable to independent validation, even when the samples are analyzed separately.

Our work shows that using a large sample size at the initial discovery phase (hundreds of subjects rather than tens), robust proteomic results can be obtained and can be effectively repeated in independent cohorts, potentially providing further clinical generalization. Robust replication is demonstrated with proteins specific to gender (that can be used for quality check of the data), proteins correlating with clinical parameters and biochemical measures relevant to obesity, and proteins associating in peripheral circulation. Through the availability of an extensive dataset of about 1500 plasma samples, preserved demographic phenotype is shown in two independent cohorts from two continents. Such practice in the proteomic field may increase the chances that identified biomarkers translate to other populations and are of clinical utility. The definition and practical demonstration of “rectangular” studies in proteomics therefore constitute important milestones, enabled by the developments and maturation of MS-based proteomic technologies. Key technological enablers to those larger clinical proteomic discovery studies have been multiple in recent years. For instance, automation of proteomic sample preparation^26^ is required to ensure throughput, robustness and accuracy, together with more rapid and more sensitive MS instrumentation. Sample multiplexing, *via* mass labeling for instance, could also represent an important asset^8^. Improvement and automation of data storage, processing, and analysis are also mandatory. All those elements, plus others, when combined, are indeed expected to empower MS-based proteomics for the discovery of novel and robust clinical biomarkers. In the past few years, we have developed a scalable and integrated proteomic pipeline^8^. We have deployed this workflow to measure a large number of plasma samples from obese and overweight individuals (*i.e*., C2A/B)^5,8^, and have herein further demonstrated its robustness and potential in clinical research with the analysis of an additional independent cohort (*i.e*., C1).

Importantly, we demonstrate the overall conservation of proteome variability (and stability) between two independent cohorts of obese and overweight individuals. This variability could explain in part why validation of biomarkers has not been always successful for a number of proteomic studies, often underpowered^7,27,28^. The next step would be to evaluate the reproducibility across laboratories. This important point is being currently investigated by different groups^29^, but would also need to be evaluated using large numbers of samples. Mann and co-workers recently showed that in general plasma protein levels tend to vary more between subjects than over the time in each subject following a dietary intervention^4^. Importantly when studying independent cohorts, not only the protein data vary between the groups, but also the confounding effects. When studying associations between the proteomes and main confounding effects such as age and gender, we again highlighted a remarkable consistency in these effects between the datasets.

Obesity is a complex disease and encompasses diverse phenotypes associated with, for instance, a range of different degrees of inflammation and multiple associated comorbidities. Analyzing populations of obese individuals for biomarker identification (either for diagnostic, prognostic, or research purposes) is therefore a very challenging task. Our findings, replicated in two independent cohorts, indicate the robustness of our results, even more when considering the inherent and noticeable differences of the cohorts, with one (*i.e*., C1) made of more extreme obese patients, from a clinical practice, than the other one (*i.e*., C2), obtained from a randomized control trial. Here again, the large sample size of the cohorts (several hundreds of patients in each) allowed generating reproducible observations.

The diagnostic accuracy of current biomarkers in the field of obesity has still not reached accuracies high enough to be used in the clinics^30^. Among current biomarkers used in obesity, there are those that aim at the early identification of obesity, its treatment definition, the assessment of the risks of complications, and the evaluation of its degree of severity. For instance, adipokines, such as leptin, TNF-alpha and resistin, have a pro-inflammatory role and are associated with an increased risk of obesity comorbidities such as T2D and cardiovascular diseases^30^. Adiponectin and omentin, on the other hand, have an anti-inflammatory and cardioprotective role, as well as being linked to glycemic control^30^. Markers for weight loss in obese populations such as CRP, SHBG, SAA proteins, members of the apolipoprotein family and of the complement pathway have been previously reported by us and others^4,5^. Some of these markers have been also associated with clinical parameters linked with obesity. In the current study, PRAP1 showed a strong positive correlation with fasting insulin levels (Fig. 3) and triglycerides (Supplementary Material, Supp. Fig. S4M,N). Those newly revealed associations represent new findings that should deserve further investigations. Taken together, those markers may enable early identification, proper treatment and follow-up of a number of co-morbidities associated with obesity, such as insulin resistance, its severity, and dyslipedemia.

While we demonstrated the application of untargeted MS-based shotgun proteomics for the discovery and replication of plasma biomarkers in independent cohorts, one limitation of our study was the still limited proteome coverage obtained. The presented data were obtained with hybrid linear ion trap-Orbitrap (LTQ-OT) Elite mass spectrometers. From our recent experience, with the latest generation of Tribrid Orbitrap mass spectrometers^31^ for instance, the human plasma proteome coverage can be drastically improved, which is expected to support the deployment of our and other untargeted MS-based proteomic strategies in clinical research. Additionally, we undertook a stringent filtering approach and discarded a number of proteins based on data missingness (without performing imputation). While there are some recommendations^32,33^ in other omics fields (*e.g*., transcriptomics and metabolomics), there is still to date, very little benchmarks and guidelines in proteomics to account for and adequately impute missing data^34^.

Blood proteomes can provide complementary information to traditional clinical measurements. Proteomes closely relate to the true physiological state of an individual. Proteins, considered in biological systems and contexts, can even better offer therapeutic targets and/or support clinical decision-making. Proteomic profiles can give information at different levels that are relevant to both the healthy state and disease status, *e.g*., obesity/overweight conditions. We showed the plasma proteome to be representative of diverse clinical parameters together. Herein, we demonstrated with real data, the robustness of MS measurements of the plasma proteome. Proteomic analysis of two large independent cohorts gave replicable results and provided insights into the obesity phenotype. From these results, we believe that in a foreseeable future, profiles and/or panels of plasma or serum protein markers would be available, that can be readily measured in the clinics.

## Experimental Procedures

### Experimental Design and Statistical Rationale

#### Description of cohorts

The cohorts, which were compared in this work, were from the Weight Management Clinical program of The Ottawa Hospital (C1) and the DiOGenes study (C2) at baseline (C2A) and after weight loss and weight maintenance (C2B). The C2 cohort and the subject and sample selection have been previously described^3,9^.

C1 was initially composed of 2383 overweight and obese patients, who underwent a meal replacement program. Patients were monitored for up to 36 weeks, checking their weight, height, blood pressure, at every visit. At week 1, they received a battery of biochemistry tests, from lipid profile to fasting insulin, and blood glucose determination.

Patients began the program between September 1993 and May 2013 and completed it between 1994 and 2014. However, *de facto*, plasma samples in this study were only from subjects followed at The Ottawa Hospital between 2003 and 2013.

Exclusion criteria before enrollment included important weight loss before the program, autoimmune disease, cancer, and previous bariatric surgery. Patients were also excluded after being admitted to the program and throughout its duration according to the following criteria:

- Non-compliance: admitting not following the meal replacement program, delayed taking the product, eating meals or not taking the product, bulimia, binge eating, extended fasting; Non-compliance with exercise, very low energy expenditure, wheelchair bound; High number of absences of the monitoring appointments.
- Taking anti-diabetic drug and drugs to control glucose, stimulant drugs, diet pills, psychotropics, antidepressants, antipsychotic or anti-epileptic drugs, or immunosuppressant drugs.
- Hospitalization and surgeries (gastroplasty, intestinal bypass, bariatric surgery, and others such as angioplasty, inguinal hernia repair, hysterectomy).
- Complications such as thyroid problems (suppressed or elevated thyroid stimulating hormone (TSH), goitre), severe insulin resistance, peripheral oedema, inflammatory bowel disease, Crohn’s disease, blindness, renal failure, meningitis, otitis, high stress levels, severe learning and memory problems, traumatic brain injury.

After exclusion, 2032 patients remained in the study, *i.e*., 1448 females and 584 males.

Questionnaires assessed their physical activity, planned and current, scores and barriers to exercise, as well as smoking status (current and previous), alcohol intake (heavy drinker, amount, problem with alcohol intake), depression, drugs, and medications taken (such as lowering or increasing blood pressure medication, lipid lowering agents, hypertension, topical corticosteroids, beta blockers). For women cyclic, menstrual status and menopause comments were recorded.

Before the MS-based proteomic analysis, additional exclusion *criteria* were considered such as pregnancy during study, compliance to weight loss protocol, plasma samples taken under fasting conditions, uses of drug affecting weight, abnormal thyroid function and type 1 diabetes. The key *criterion* was the availability of the plasma samples.

For C2A and C2B, the proteomic results were previously reported^3^. One collection center previously shown to present a clear deviation from the other centers^3^ was excluded from the C2 cohort considered in this study. The choice of N was based on the largest available number of samples.

#### Dataset completeness of proteomic datasets

C1 comprised 577 plasma samples, for whom 507 proteins were measured (data matrix completeness of 66.5%). Among these, 154 proteins contained no missing values. In our filtered dataset used herein, 299 proteins contained at most 30% missing values, giving a total data matrix completeness of 94.8%. C2A, on the other hand comprised 425 plasma samples, for whom 364 proteins were measured (data matrix completeness of 58.4%), 182 of which have a maximum of 30% missing values giving a final filtered dataset completeness of 95.4%. For C2B, 373 proteins were measured in 429 plasma samples (data matrix completeness of 58.4%), of which 182 contained at most 30% of missing values, giving a final filtered dataset completeness of 95.4%. Figures of data completeness can be found in the Supplementary Material (Supp. Fig. S8). In total, 327 proteins were found in common between C1, C2A and C2B, and 179 proteins among them had at most 30% of missing values. These protein lists and the data tables can be found in the Supplementary Material.

### Materials, Sample Preparation and MS Analysis

#### Materials

Iodoacetamide (IAA), tris(2-carboxyethyl) phosphine hydrochloride (TCEP), triethylammonium hydrogen carbonate buffer (TEAB) 1M pH = 8.5, sodium dodecyl sulfate (SDS), and LACB from bovine milk were purchased from Sigma (St. Louis, MO, USA). Formic acid (FA, 99%) was from BDH (VWR International Ltd., Poole, UK). Hydroxylamine solution 50 *wt*% in H_2_O (99.999%) was acquired from Aldrich (Milwaukee, WI, USA). Water (18.2 MΩ·cm at 25 °C) was obtained from a Milli-Q apparatus (Millipore, Billerica, MA, USA) and acetonitrile from BDH. Trifluoroacetic acid Uvasol® was sourced from Merck Millipore (Billerica, MA, USA). The sixplex tandem mass tags (TMTs) were purchased from Thermo Scientific (Rockford, IL, USA). Sequencing grade-modified trypsin was procured from Promega (Madison, WI, USA). For immuno-affinity depletion of 14 abundant human proteins, multiple affinity removal system (MARS) columns, Buffer A, and Buffer B were obtained from Agilent Technologies (Wilmington, DE, USA). Oasis HLB cartridges (1cc, 30 mg) were acquired from Waters (Milford, MA, USA) and strong cation-exchange (SCX) solid-phase extraction (SPE) cartridges from Phenomenex (Torrance, CA, USA).

#### Sample preparation

From 25 µL of each plasma sample (diluted in 75 µL of Buffer A containing 0.0134 mg/mL LACB and filtered with 0.22 µm filter plate from Millipore), 14 abundant plasma proteins were removed, following the manufacturer instructions, with MARS columns and high performance (HP) LC systems (Thermo Scientific, San Jose, CA, USA) equipped with an HTC-PAL (CTC Analytics AG, Zwingen, Switzerland) fraction collectors. After immunodepletion, samples were snap-frozen. Buffer exchange was performed with Strata-X 33 u Polymeric reversed-phase (RP) (30 mg/1 mL) cartridges mounted on a 96-hole holder and a *vacuum* manifold, all from Phenomenex as previously described^8^. Samples were subsequently evaporated with a *vacuum* centrifuge (Thermo Scientific) and stored at −80 °C. Reduction, alkylation, digestion, TMT sixplex labeling, and SPE sample purification (Oasis HLB and SCX) were performed on a 4-channels Microlab Star liquid handler (Hamilton, Bonaduz, Switzerland) according to a previously reported and validated protocol^8^. Briefly, each lyophilized sample was dissolved in 95 µL of TEAB 100 mM and 5 µL of SDS 2%. Next, a volume of 5.3 µL TCEP 20 mM was added and incubation was performed for 1 h at 55 °C. A volume of 5.5 µL IAA 150 mM was added (incubation for 1 h in the dark). Following this step, enzymatic digestion was performed by addition of 10 µL trypsin at 0.25 µg·µL^−1^ in TEAB 100 mM and incubation overnight at 37 °C. TMT labelling was performed by addition of 0.8 mg sixplex TMT reagent in 41 µL CH_3_CN (incubation for 1 h at room temperature). After reaction, a volume of 8 µL hydroxylamine 5% in H_2_O was added to each tube to react for 15 min. Samples from a given sixplex TMT experiment were pooled together in a new tube (each TMT experiment included four samples and two pool plasma samples which were used as biological references for relative quantification of proteins). Pooled samples were further purified with Oasis HLB cartridges followed by SCX SPE. The purified pooled sixplex TMT-labeled samples were then evaporated to dryness before storage at −80 °C.

#### LC-MS/MS analysis

The samples were dissolved in 500 µL H_2_O/CH_3_CN/FA 96.9/3/0.1 for RP-LC-MS/MS. LC-MS/MS was performed on two identical systems composed of a hybrid LTQ-OT Elite and an Ultimate 3000 RSLC nano system (Thermo Scientific). Proteolytic peptides (injection of 5 µL of sample) were trapped on an Acclaim PepMap 75 µm × 2 cm (C18, 3 µm, 100 Å) pre-column and separated on an Acclaim PepMap RSLC 75 µm × 50 cm (C18, 2 µm, 100 Å) column (Thermo Scientific) coupled to a stainless steel nanobore emitter (40 mm, OD 1/32”) mounted on a Nanospray Flex Ion Source (Thermo Scientific). The analytical separation was run for 150 min using a gradient that reached 30% of CH_3_CN after 140 min and 80% of CH_3_CN after 150 min at a flow rate of 220 nL/min. For MS survey scans, the OT resolution was 120000 (ion population of 1 × 10^6^) with an *m*/*z* window from 300 to 1500. For MS/MS with higher-energy collisional dissociation at 35% of the normalized collision energy, ion population was set to 1 × 10^5^ (isolation width of 2), with a resolution of 15000, first mass at *m*/*z* = 100, and a maximum injection time of 250 ms in the OT. A maximum of 10 precursor ions (most intense) were selected for MS/MS. Ions with 1+ and unassigned charge-states were rejected from the MS/MS analysis. Dynamic exclusion was set for 60 s within a ± 5 ppm window. A lock mass of m/z = 445.1200 was used. Each sample was analyzed in duplicate once on each of the two independent but identical RP-LC-MS/MS platforms. The exact same setup and instrumentation were used for both studies (*i.e*., C1 and C2) with a 15-month interval. The mass spectrometers were calibrated every week and transfer tubes were cleaned. A complex protein digest was analyzed in triplicate on the RP-LC-MS/MS platforms to validate their performance before analysis of a batch of samples^35^. LC columns were changed every 3 weeks and the solvents freshly prepared.

### Computational and Bioinformatics Analysis

#### Computational analysis

Proteome Discoverer (version 1.4, Thermo Scientific) was used as data processing interface. Identification was performed against the human UniProtKB/Swiss-Prot database (24/07/2013 or 08/12/2014 releases) including the LACB sequence (20268 and 20194 sequences in total, respectively). Mascot (version 2.4.0 or 2.4.2, Matrix Sciences, London, UK) was used as search engine. Variable amino acid modifications were oxidized methionine, deamidated asparagine/glutamine, and sixplex TMT-labeled peptide amino terminus. Sixplex TMT-labeled lysine was set as fixed modifications as well as carbamidomethylation of cysteine. Trypsin was selected as the proteolytic enzyme, with a maximum of two potential missed cleavages. Peptide and fragment ion tolerances were set to, respectively, 10 ppm and 0.02 Da. All Mascot result files were loaded into Scaffold Q + 4.2.1 or 4.4.3 (Proteome Software, Portland, OR, USA) to be further searched with X! Tandem. Both peptide and protein FDRs were fixed at 1%, with a two unique peptide *criterion* to report protein identification. MS data quality was monitored by assessing in the samples the consistence of proteome coverage and precision and trueness of LACB relative quantification as previously reported^3^. Problematic samples were flagged (for possible removal from the following bioinformatic analysis) but no batch (*i.e*., plate) issue was detected during both studies according to such type of *criterion*^36^. Relative quantitative protein values were exported from Scaffold Q+ as Log2 of the protein ratio fold-changes with respect to their measurements in the biological reference, *i.e*., mean Log2 values after isotopic purity correction but without normalization applied between samples and experiments. The biological references were pools of individual plasma samples.

#### Bioinformatic analysis

Statistical analysis was performed using the statistical language and environment R^37^. In particular, the function rcorr form the Hmisc package and the function knnImputation from the DMwR package were used.

Clinical variables were presented as mean ± SD, and protein measurements in Log2 ratios respective to pool of samples. For the comparison of the SD of proteins (Fig. 2B), data was filtered with a 5% maximum missing value threshold and data was imputed using the weighted KNN imputation algorithm^38^, method weighted average (Euclidean distance to neighbours being used as weights), *k* = 15, where this value was chosen as it was the number of neighbours that minimized the RSS average of 1000 iterations of the complete datasets having randomly removed 5 percent of their data values, performed KNNImputation for *k* ranging from 1 to 50 neighbours (plots shown in Supplementary Material, Supp. Fig. S9). In order to determine gender-specific proteins (Fig. 2F-H), proteins with a maximum of 30% missing values were kept and no imputation was performed. This increased tolerance was applied in order to include all proteins that may mostly be present in one of the genders. For the identification of potential confounding factors between plasma proteins levels and clinical variables, we used linear models as follows: the linear models used to determine the relationship between plasma protein levels and clinical variables (results shown in Tables 2–4 and in Supp. Figs S1 and S2) were described using the following relationship: (1) Prot_i ~ Age + Gender + BMI, for every protein^39^. *P*-values were adjusted for multiple testing using the Benjamini-Hochberg correction^40^. The Spearman correlations shown in Fig. 3 and Supp. Figs S3 and S4, were computed between clinical variables Clinical_Variable_j and e_i, where e_i are the residuals of model (2) Prot_i ~ Age + Gender. *P*-values were corrected using the Benjamini-Hochberg (BH). Significance was set at BH-corrected *p*-values less than 0.05.

### Ethics

The studies were approved by local ethical committees in the respective countries. For C1: Ottawa Health Science Network Research Ethics Board and for C2: the Medical Ethics Committee of the University Hospital Maastricht and Maastricht University, the Netherlands; the Committees on Biomedical Research Ethics for the Capital region of Denmark, Denmark; the Suffolk Local Research Ethics Committee, United Kingdom; the University of Crete Ethics Committee, Greece; the Ethics Commission of the University of Potsdam, Germany; the Research Ethics Committee at the University of Navarra, Spain; the Ethical Committee of the Institute of Endocrinology, Czech Republic; and the Ethical Committee to the National Transport Multiprofile Hospital in Sofia, Bulgaria. For both cohorts, the Cantonal Ethics Committee for Research on Human Beings, Vaud, Switzerland approved the study protocol to be performed at the Nestlé Institute of Health Sciences. All study participants (and for the children who are not legally to sign also the parents) signed an informed consent document after verbal and written instructions, and according to local legislation. All methods were performed in accordance with the relevant guidelines and regulations.

## Electronic supplementary material

Supplementary Dataset C1

Supplementary Dataset C2A

Supplementary Dataset S3

Supplementary Material

## Data Availability

The MS proteomic data have been deposited to the ProteomeXchange Consortium *via* the PRIDE^41^ partner repository with the dataset identifier PXD009350 (project 10.6019/PXD009350) for C1. The data were previously deposited with the dataset identifier PXD005216 for C2. The datasets used and/or analyzed during the current study are available from the corresponding author upon request and ethical approval.

## References

[1] Geyer PE, Holdt LM, Teupser D, Mann M (2017). Revisiting biomarker discovery by plasma proteomics. Mol Syst Biol.

[2] Liu Y (2015). Quantitative variability of 342 plasma proteins in a human twin population. Mol Syst Biol.

[3] Cominetti O (2016). Proteomic biomarker discovery in 1000 human plasma samples with mass spectrometry. J Proteome Res.

[4] Geyer PE (2016). Proteomics reveals the effects of sustained weight loss on the human plasma proteome. Mol Syst Biol.

[5] Oller Moreno, S. *et al*. The differential plasma proteome of obese and overweight individuals undergoing a nutritional weight loss and maintenance intervention. *Proteomics Clin Appl* **12**, 1600150 (2018). 10.1002/prca.201600150 28371297

[6] Martens L (2013). Bringing proteomics into the clinic: the need for the field to finally take itself seriously. Proteomics Clin Appl.

[7] Hernández B, Parnell A, Pennington SR (2014). Why have so few proteomic biomarkers “survived” validation? (Sample size and independent validation considerations). Proteomics.

[8] Dayon Loïc, Núñez Galindo Antonio, Corthésy John, Cominetti Ornella, Kussmann Martin (2014). Comprehensive and Scalable Highly Automated MS-Based Proteomic Workflow for Clinical Biomarker Discovery in Human Plasma. Journal of Proteome Research.

[9] Larsen TM (2010). The diet, obesity and genes (Diogenes) dietary study in eight European countries - a comprehensive design for long-term intervention. Obes Rev.

[10] Visser M, Bouter LM, McQuillan GM, Wener MH, Harris TB (1999). Elevated c-reactive protein levels in overweight and obese adults. JAMA.

[11] Randall Sarah A., McKay Matthew J., Pascovici Dana, Mahon Kate, Horvath Lisa, Clarke Stephen J., Molloy Mark P. (2012). Remarkable temporal stability of high-abundance human plasma proteins assessed by targeted mass spectrometry. PROTEOMICS - Clinical Applications.

[12] Khera A (2005). Race and gender differences in C-reactive protein levels. J Am Coll Cardiol.

[13] Lakoski SG (2006). Gender and C-reactive protein: data from the Multiethnic Study of Atherosclerosis (MESA) cohort. Am Heart J.

[14] Qasim AN (2011). Gender differences in the association of C-reactive protein with coronary artery calcium in type-2 diabetes. Clin Endocrinol (Oxf).

[15] Piéroni L (2003). Interpretation of circulating C-reactive protein levels in adults: body mass index and gender are a must. Diabetes Metab.

[16] McConnell JP (2002). Gender differences in C-reactive protein concentrations-confirmation with two sensitive methods. Clin Chem Lab Med.

[17] Rifai N, Ridker PM (2003). Population distributions of C-reactive protein in apparently healthy men and women in the United States: implication for clinical interpretation. Clin Chem.

[18] Enroth S, Johansson A, Enroth SB, Gyllensten U (2014). Strong effects of genetic and lifestyle factors on biomarker variation and use of personalized cutoffs. Nat Commun.

[19] Kei AA, Filippatos TD, Tsimihodimos V, Elisaf MS (2012). A review of the role of apolipoprotein C-II in lipoprotein metabolism and cardiovascular disease. Metabolism - Clinical and Experimental.

[20] Curran AM (2017). Sexual dimorphism, age, and fat mass are key phenotypic drivers of proteomic signatures. J Proteome Res.

[21] Silliman CC (2012). Proteomic analyses of human plasma: Venus versus Mars. Transfusion.

[22] Fernandez-Real JM (2002). Serum corticosteroid-binding globulin concentration and insulin resistance syndrome: a population study. J Clin Endocrinol Metab.

[23] Sen, P. K. & Singer, J. M. *Large Sample Methods in Statistics: An Introduction with Applications*. (Taylor & Francis, 1994).

[24] Davison, A. C. & Hinkley, D. V. *Bootstrap Methods and their Application*. (Cambridge University Press, 2013).

[25] Evans, M. J. & Rosenthal, J. S. *Probability and Statistics: The Science of Uncertainty*. (W. H. Freeman, 2004).

[26] Guh DP (2009). The incidence of co-morbidities related to obesity and overweight: a systematic review and meta-analysis. BMC Public Health.

[27] Mischak H (2015). Epidemiologic design and analysis for proteomic studies: a primer on -omic technologies. Am J Epidemiol.

[28] Robin X (2011). pROC: an open-source package for R and S+ to analyze and compare ROC curves. BMC Bioinformatics.

[29] Collins BC (2017). Multi-laboratory assessment of reproducibility, qualitative and quantitative performance of SWATH-mass spectrometry. Nature Communications.

[30] Katsareli EA, Dedoussis GV (2014). Biomarkers in the field of obesity and its related comorbidities. Expert Opin Ther Targets.

[31] Lan J (2018). Systematic evaluation of the use of human plasma and serum for mass-spectrometry-based shotgun proteomics. J Proteome Res.

[32] Aittokallio T (2010). Dealing with missing values in large-scale studies: microarray data imputation and beyond. Brief Bioinformatics.

[33] Wei, R. *et al*. Missing value imputation approach for mass spectrometry-based metabolomics data. *Sci Rep* **8**, 663 (2018). 10.1038/s41598-017-19120-0 PMC5766532 29330539

[34] Lazar C, Gatto L, Ferro M, Bruley C, Burger T (2016). T. Accounting for the multiple natures of missing values in label-free quantitative proteomics data sets to compare imputation strategies. J Proteome Res.

[35] Dayon L, Galindo AN, Cominetti O, Corthésy J, Kussmann M (2017). A highly automated shotgun proteomic workflow: clinical scale and robustness for biomarker discovery in blood. Methods Mol Biol.

[36] Song Xiaomin, Amirkhani Ardeshir, Wu Jemma X., Pascovici Dana, Zaw Thiri, Xavier Dylan, Clarke Stephen J., Molloy Mark P. (2016). Analytical performance of nano-LC-SRM using nondepleted human plasma over an 18-month period. PROTEOMICS.

[37] R: A language and environment for statistical computing (R Foundation for Statistical Computing, 2014).

[38] Torgo, L. *Data Mining with R: Learning with Case Studies*. (Chapman\& Hall/CRC, 2010).

[39] Chambers, J. M. *Statistical Models in S*. (CRC Press, Inc., 1991).

[40] Benjamini Y, Hochberg Y (1995). Controlling the False Discovery Rate: A Practical and Powerful Approach to Multiple Testing*J R Stat Soc*. Series B (Methodological).

[41] Vizcaíno JA (2016). 2016 update of the PRIDE database and its related tools. Nucleic Acids Res.

