# Supplementary material for: Obesity shows preserved plasma proteome in large independent clinical cohorts
Source: Sci Rep. 2018 Nov 19;8:16981. doi: 10.1038/s41598-018-35321-7 (PMC6242904; doi:10.1038/s41598-018-35321-7)
Supplement: Supplementary file 4 — Supplementary Material [file 41598_2018_35321_MOESM4_ESM.pdf]

Supplementary Material

Obesity shows preserved plasma proteome in large independent clinical cohorts

Ornella Cominetti[1], Antonio Núñez Galindo[1, +], John Corthésy[1, 2, +], Armand Valsesia[3, +], Irina Irincheeva[3, ±], Martin Kussmann[1, #], Wim H.M. Saris[4], Arne Astrup[5], Ruth McPherson[6], Mary-Ellen Harper[7], Robert Dent[8], Jörg Hager[3], and Loïc Dayon[1, *]

[1] Proteomics, Nestlé Institute of Health Sciences, Lausanne, Switzerland
[2] Nutrition Analytics, Nestlé Institute of Health Sciences, Lausanne, Switzerland
[3] Nutrition and Metabolic Health, Nestlé Institute of Health Sciences, Lausanne, Switzerland
[4] NUTRIM, School for Nutrition, Toxicology and Metabolism, Department of Human Biology, Maastricht University Medical Centre, Maastricht, The Netherlands
[5] Department of Nutrition, Exercise and Sports, Faculty of Science, University of Copenhagen, Denmark
[6] Ruddy Canadian Cardiovascular Genetics Centre, University of Ottawa Heart Institute, Ottawa, Canada
[7] Department of Biochemistry, Microbiology and Immunology, Faculty of Medicine, University of Ottawa, Ottawa Canada
[8] Ottawa Hospital Weight Management Clinic, The Ottawa Hospital, Ottawa, Canada

[+] Contributed equally
[±] Current address: Clinical Trial Unit, University of Bern, Switzerland
[#] Current address: The Liggins Institute, University of Auckland, Auckland, New Zealand
[*] Corresponding author: Nestlé Institute of Health Sciences, EPFL Innovation Park, Bâtiment H, 1015 Lausanne, Switzerland; Email: loic.dayon@rd.nestle.com, Phone: +41 21 632 6114, Fax: +41 21 632 6499

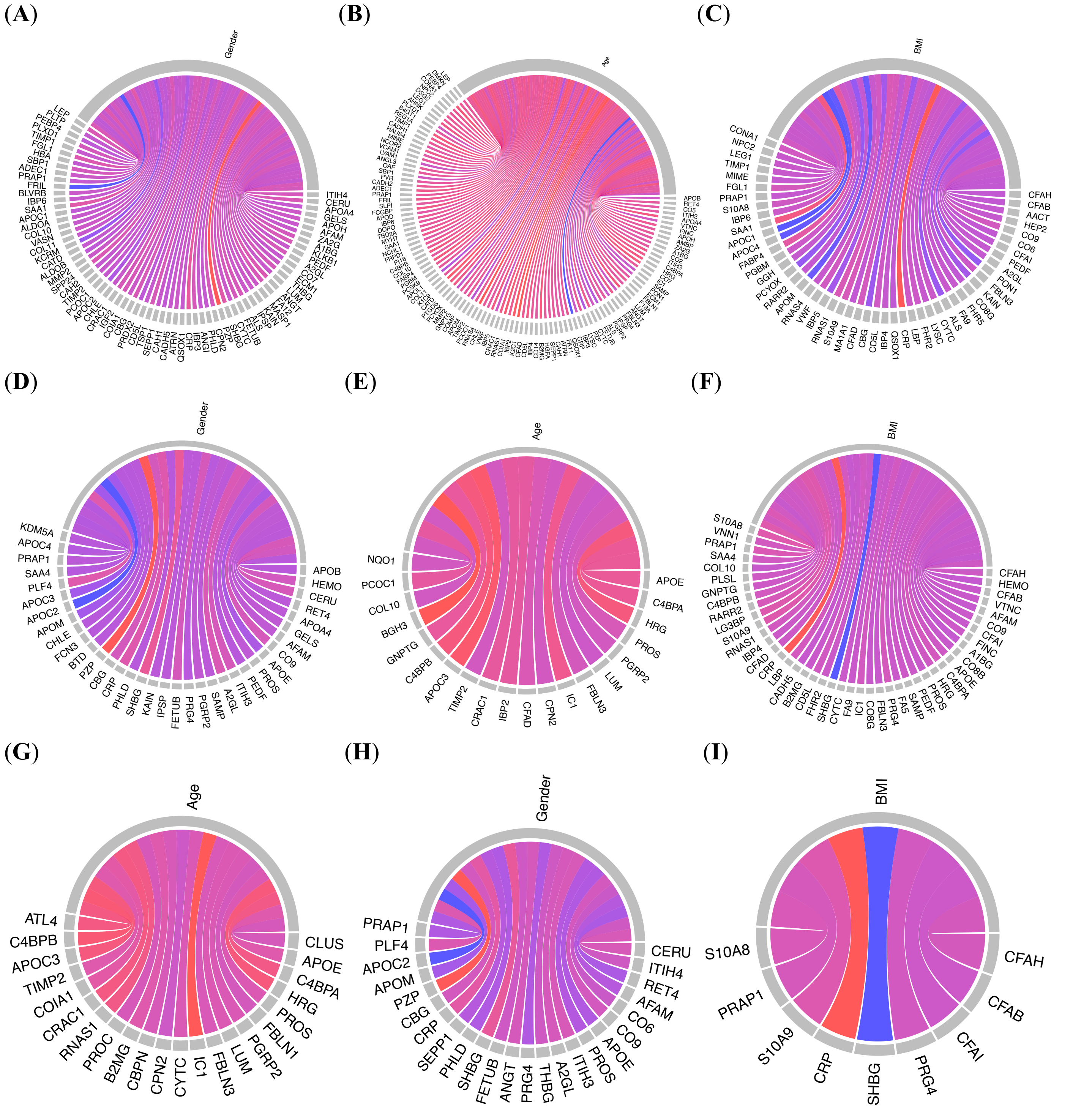

Figure S1. Chord diagrams depicting common significant covariates for proteins (adjusted p-values ≤ 0.05) in (**A-C**) C1, (**D-F**) C2A and (**G-I**) C2B with respect to (left) gender, (middle) age and (right) BMI. Color code corresponds to the average of the estimates for each cohort, with blue corresponding to the most negative estimate for all the proteins with respect to gender, age or BMI, and red corresponding to the most positive estimate for all the proteins with respect to each confounder.

(**A**) (**B**) (**C**)

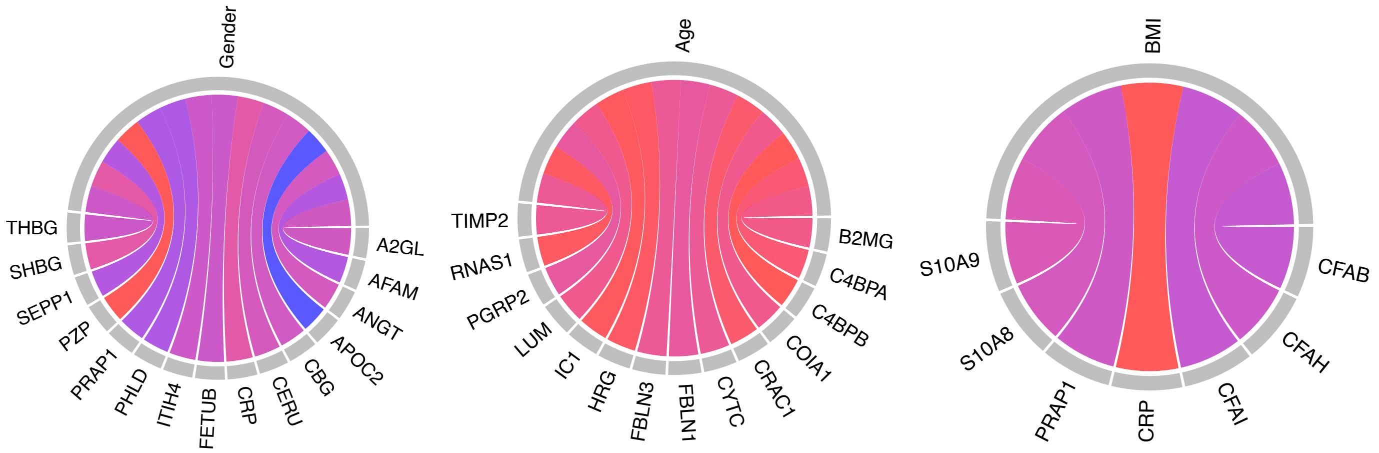

Figure S2. Chord diagrams depicting common significant covariates for proteins (adjusted p-values ≤ 0.05) in the three cohorts, *i.e.*, C1, C2A and C2B with respect to (**A**) gender, (**B**) age and (**C**) BMI. There are 11 significant associations for gender, nine for age and seven for BMI. Color code corresponds to the average of the estimates for each cohort, with blue corresponding to the most negative estimate for all the proteins with respect to gender, age or BMI, and red corresponding to the most positive estimate for all the proteins with respect to each confounder.

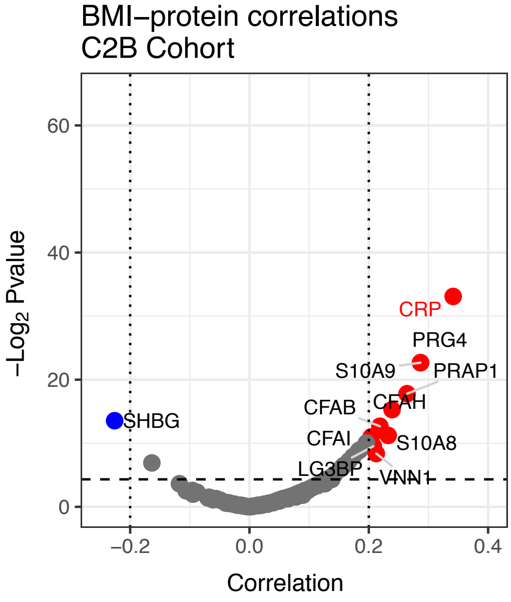

Figure S3. Volcano plot of BMI-protein correlations for C2B. The horizontal dashed line in each plot represents the threshold of significance of adjusted p-value ≤ 0.05 and the vertical dotted lines represent the limits of -0.2 and 0.2 of the Spearman correlation coefficients. Only the names of the proteins significantly regulated are shown, and they are colored if they are significant also for the cohorts C1 and C2A (see **Fig. 4** in the main manuscript). Red corresponds to positive correlated proteins (right) and in blue the negatively correlated proteins (left).

From **Supp. Fig. S3** and **Fig. S4**, we observed that the BMI-protein correlation plots between C2A and C2B were remarkably similar, which must be due to the fact that they corresponded to the same subjects before and after weight loss and management. Even if individuals undergo such type of intervention, their protein values remained associated to their clinical parameters in a similar fashion.

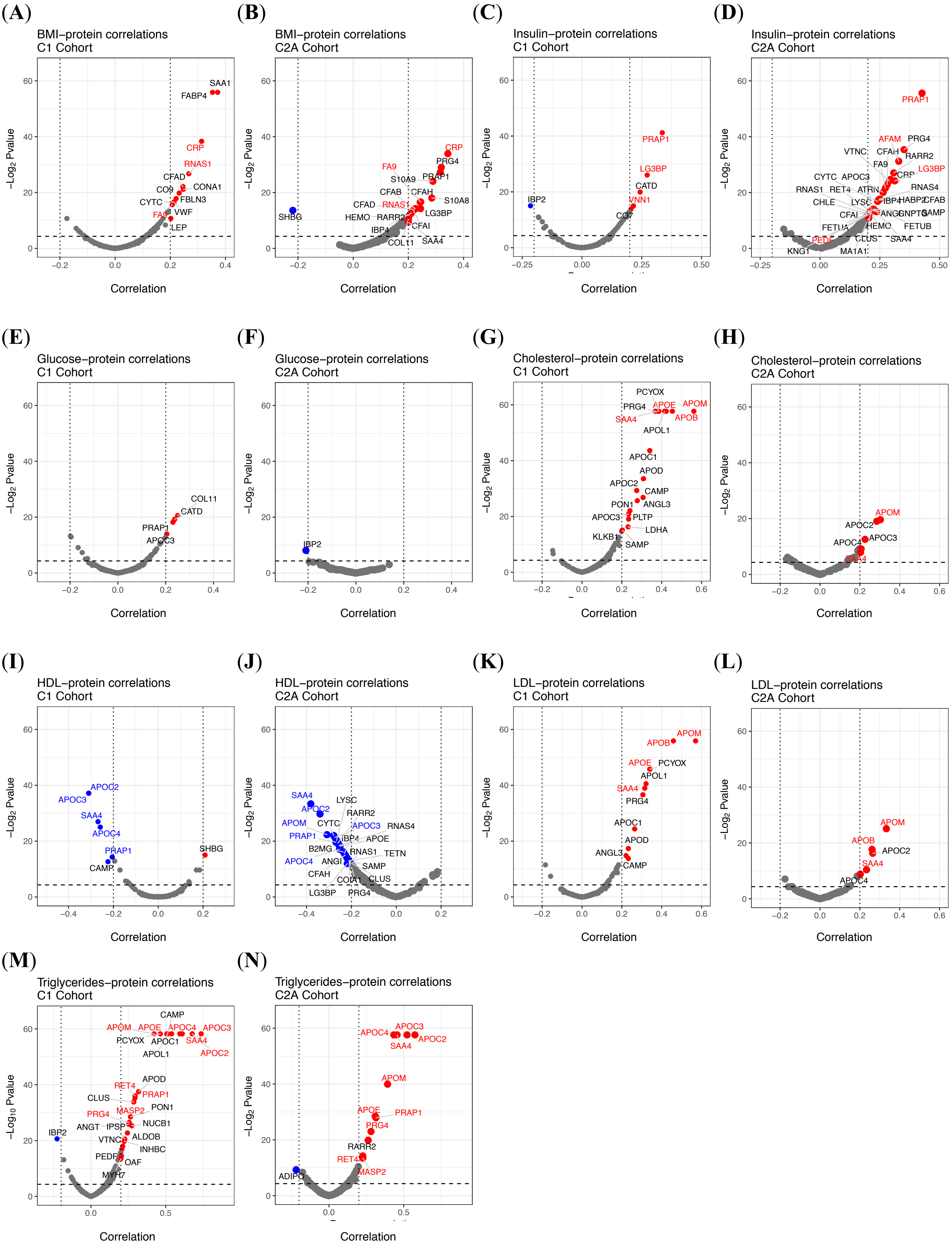
(A)
BMI−protein correlations
C1 Cohort
SAA1
FABP4
CRP
RNAS1
CFAD
CO9
CONA1
FBLN3
CYTC
VWF
FA9
LEP
(B)
BMI−protein correlations
C2A Cohort
CRP
PRG4
FA9
S10A9
PRAP1
CFAB
CFAH
S10A8
CFAD
RNAS1
SHBG
HEMO
RARR2
LG3BP
IBP4
CFAI
COL11
SAA4
(C)
Insulin−protein correlations
C1 Cohort
PRAP1
LG3BP
CATD
IBP2
VNN1
CO7
(D)
Insulin−protein correlations
C2A Cohort
PRAP1
AFAM
PRG4
VTNC
CFAH
RARR2
FA9
LG3BP
CYTC
APOC3
CRP
RNAS1
RET4
ATRN
RNAS4
CHLE
LYSC
IBP4
HABP2
CFAB
CFAI
ANGT
GNPTG
SAMP
FETUA
HEMO
FETUB
PEDF
CLUS
SAA4
KNG1
MA1A1
(E)
Glucose−protein correlations
C1 Cohort
COL11
CATD
PRAP1
APOC3
(F)
Glucose−protein correlations
C2A Cohort
IBP2
(G)
Cholesterol−protein correlations
C1 Cohort
PCYOX
PRG4
APOE
APOM
SAA4
APOB
APOL1
APOC1
APOD
APOC2
CAMP
PON1
ANGL3
APOC3
PLTP
LDHA
KLKB1
SAMP
(H)
Cholesterol−protein correlations
C2A Cohort
APOM
APOC2
APOC4
APOC3
SAA4
(I)
HDL−protein correlations
C1 Cohort
APOC2
APOC3
SAA4
APOC4
PRAP1
SHBG
CAMP
(J)
HDL−protein correlations
C2A Cohort
SAA4
LYSC
APOC2
RARR2
APOM
CYTC
APOC3
RNAS4
PRAP1
IBP4
APOE
B2MG
RNAS1
TETN
APOC4
ANGT
SAMP
CFAH
COIA1
CLUS
LG3BP
PRG4
(K)
LDL−protein correlations
C1 Cohort
APOM
APOB
APOE
PCYOX
APOL1
SAA4
PRG4
APOC1
APOD
ANGL3
CAMP
(L)
LDL−protein correlations
C2A Cohort
APOM
APOB
APOC2
SAA4
APOC4
(M)
Triglycerides−protein correlations
C1 Cohort
CAMP
APOM
APOE
APOC4
APOC3
PCYOX
APOC1
SAA4
APOL1
APOC2
APOD
RET4
CLUS
PRAP1
PRG4
MASP2
PON1
ANGT
IPSP
NUCB1
IBP2
ALDOB
VTNC
INHBC
PEDF
OAF
MYH7
(N)
Triglycerides−protein correlations
C2A Cohort
APOC4
APOC3
APOC2
SAA4
APOM
APOE
PRAP1
PRG4
RARR2
RET4
MASP2
ADIPO
−Log₂ Pvalue
−Log₁₀ Pvalue
Correlation

Figure S4. Clinical variable-protein correlations for C1 and C2A after removing effect from gender and age, shown as volcano plots. (**A**)-(**B**): BMI [kg/m^2], (**C**)-(**D**): fasting insulin [mIU/L derived], (**E**)-(**F**): fasting glucose [mmol/L derived], (**G**)-(**H**): cholesterol, fasting [mmol/L], (**I**)-(**J**): HDL, fasting [mmol/L], (**K**)-(**L**): LDL, fasting [mmol/L], and (**M**)-(**N**) triglycerides, fasting [mmol/L]. The horizontal dashed line in each plot represents the threshold of significance of adjusted p-value ≤ 0.05 and the vertical dotted lines represent the limits of -0.2 and 0.2 of the Spearman correlation coefficients. Only the names of the proteins significantly regulated are shown, and they are colored if they are significant for both cohorts in the original analysis without considering confounders, in **Fig. 4**. Red corresponds to positive correlated proteins (*i.e.*, on the right) and in blue are depicted the negatively correlated proteins (*i.e.*, on the left).

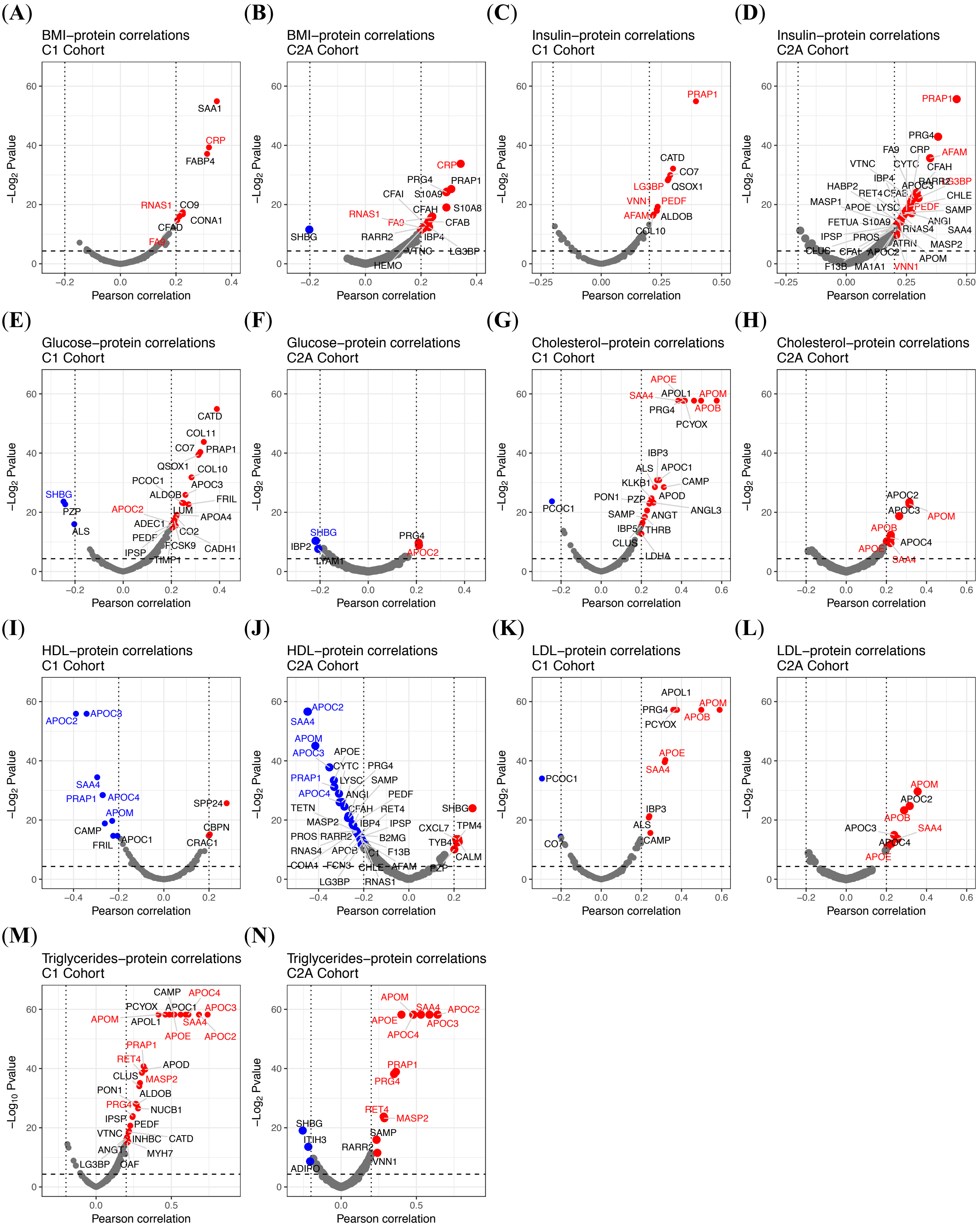

(A)
BMI–protein correlations
C1 Cohort
SAA1
CRP
FABP4
RNAS1
CO9
CONA1
CFAD
FA9
(B)
BMI–protein correlations
C2A Cohort
CRP
PRG4
PRAP1
CFAI
S10A9
CFAH
S10A8
RNAS1
FA9
CFAB
SHBG
RARR2
IBP4
VTNC
LG3BP
HEMO
(C)
Insulin–protein correlations
C1 Cohort
PRAP1
CATD
CO7
LG3BP
QSOX1
VNN1
PEDF
AFAM
ALDOB
COL10
(D)
Insulin–protein correlations
C2A Cohort
PRAP1
PRG4
FA9
CRP
AFAM
VTNC
CYTC
CFAH
IBP4
RARR2
LG3BP
HABP2
APOC3
RET4
CFAB
CHLE
MASP1
APOE
LYSC
PEDF
SAMP
FETUA
S10A9
ANGI
RNAS4
SAA4
IPSP
PROS
ATRN
MASP2
CLUS
CFAI
APOC2
APOM
F13B
MA1A1
VNN1
(E)
Glucose–protein correlations
C1 Cohort
CATD
COL11
CO7
PRAP1
QSOX1
COL10
PCOC1
APOC3
ALDOB
FRIL
SHBG
PZP
APOC2
LUM
APOA4
ALS
ADEC1
PEDF
CO2
FCSK9
CADH1
IPSP
TIMP1
(F)
Glucose–protein correlations
C2A Cohort
SHBG
PRG4
IBP2
APOC2
LYAM1
(G)
Cholesterol–protein correlations
C1 Cohort
APOE
SAA4
APOL1
APOM
PRG4
APOB
PCYOX
IBP3
ALS
APOC1
KLKB1
CAMP
PON1
PZP
APOD
PCOC1
SAMP
ANGT
ANGL3
IBP5
THRB
CLUS
LDHA
(H)
Cholesterol–protein correlations
C2A Cohort
APOC2
APOC3
APOM
APOB
APOC4
APOE
SAA4
(I)
HDL–protein correlations
C1 Cohort
APOC3
APOC2
SAA4
PRAP1
APOC4
SPP24
APOM
CBPN
CAMP
APOC1
FRIL
CRAC1
(J)
HDL–protein correlations
C2A Cohort
APOC2
SAA4
APOM
APOC3
APOE
CYTC
PRG4
PRAP1
LYSC
SAMP
APOC4
ANGI
PEDF
TETN
CFAH
RET4
SHBG
MASP2
IBP4
IPSP
CXCL7
TPM4
PROS
RARR2
B2MG
TYB4
RNAS4
APOB
C1
F13B
CALM
COIA1
FCN3
CHLE
AFAM
PZP
LG3BP
RNAS1
(K)
LDL–protein correlations
C1 Cohort
APOL1
APOM
PRG4
APOB
PCYOX
APOE
SAA4
PCOC1
IBP3
ALS
CO7
CAMP
(L)
LDL–protein correlations
C2A Cohort
APOM
APOC2
APOB
SAA4
APOC3
APOC4
APOE
(M)
Triglycerides–protein correlations
C1 Cohort
CAMP
APOC4
PCYOX
APOC1
APOC3
APOM
APOL1
SAA4
APOE
APOC2
PRAP1
RET4
APOD
CLUS
MASP2
PON1
ALDOB
PRG4
NUCB1
IPSP
PEDF
VTNC
INHBC
CATD
ANGT
MYH7
LG3BP
QAF
(N)
Triglycerides–protein correlations
C2A Cohort
APOM
SAA4
APOC2
APOE
APOC3
APOC4
PRAP1
PRG4
RET4
MASP2
SHBG
SAMP
ITIH3
RARR2
VNN1
ADIPO
Pearson correlation
$-\text{Log}_2$ Pvalue
$-\text{Log}_{10}$ Pvalue

Figure S5. Clinical variable-protein correlations for C1 and C2A, shown as volcano plots. (**A**)-(**B**): BMI [kg/m^2], (**C**)-(**D**): fasting insulin [mIU/L derived], (**E**)-(**F**): fasting glucose [mmol/L derived], (**G**)-(**H**): cholesterol, fasting [mmol/L], (**I**)-(**J**): HDL, fasting [mmol/L], (**K**)-(**L**): LDL, fasting [mmol/L], and (**M**)-(**N**) triglycerides, fasting [mmol/L]. *P*-values were corrected for multiplicity testing using the Benjamini-Hochberg procedure. The horizontal dashed line in each plot represents the threshold of significance of *p*-value ≤ 0.05 and the vertical dotted lines represent the limits of -0.2 and 0.2 of the Pearson correlation coefficients. Only the names of the proteins significantly regulated are shown, and they are colored if they are significant for both cohorts. Red corresponds to positive correlated proteins (*i.e.*, on the right) and in blue are depicted the negatively correlated proteins (*i.e.*, on the left).

(A) **(B)**

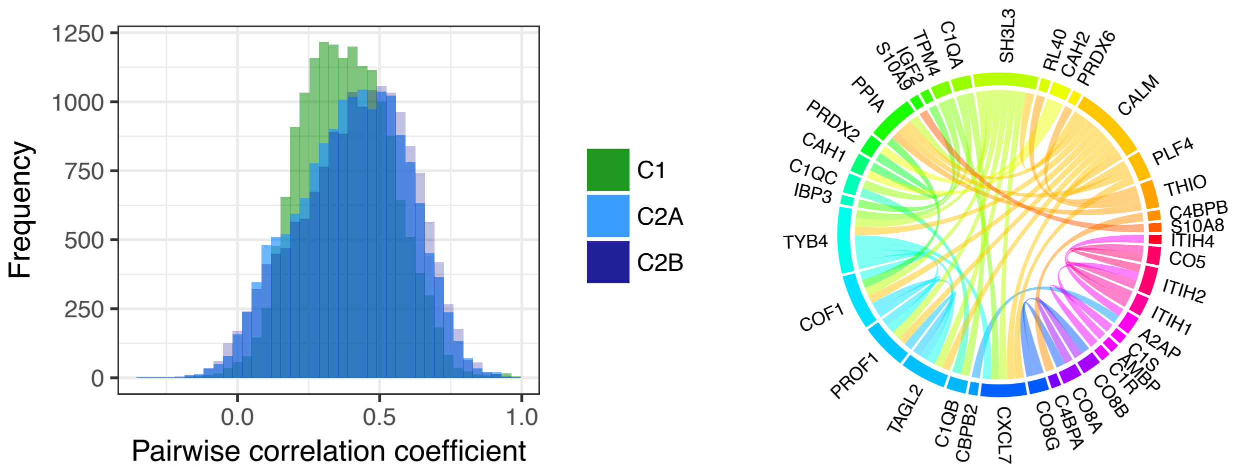

Figure S6. (**A**) Histogram of pairwise correlation coefficients for the three cohorts, C1 in green and C2 in two shades of blue: C2A in light blue and C2B in dark blue. (**B**) Chord diagram representing significant correlations between pairs of proteins in C2B (Spearman $R > 0.85$).

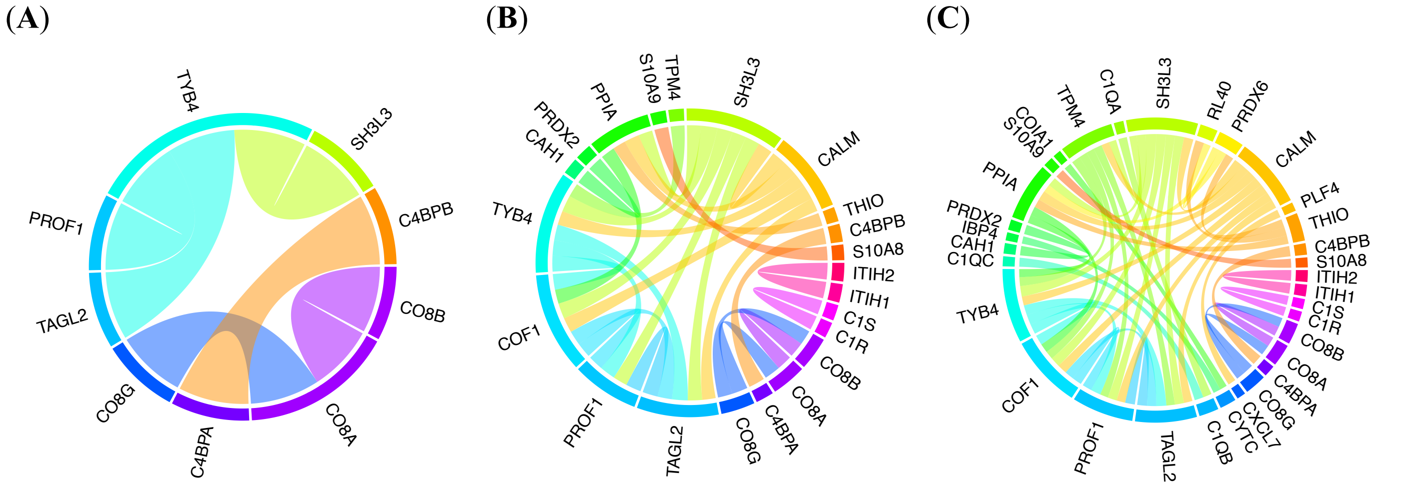

Figure S7. Common inter-protein relationships between C1 and C2A and C2B. For decreasing stringency: thresholds for Spearman correlation coefficients of (**A**) 0.90, (**B**) 0.85 and (**C**) 0.80. Number of significant pairs were (**A**) 6, (**B**) 27, and (**C**) 43, respectively.

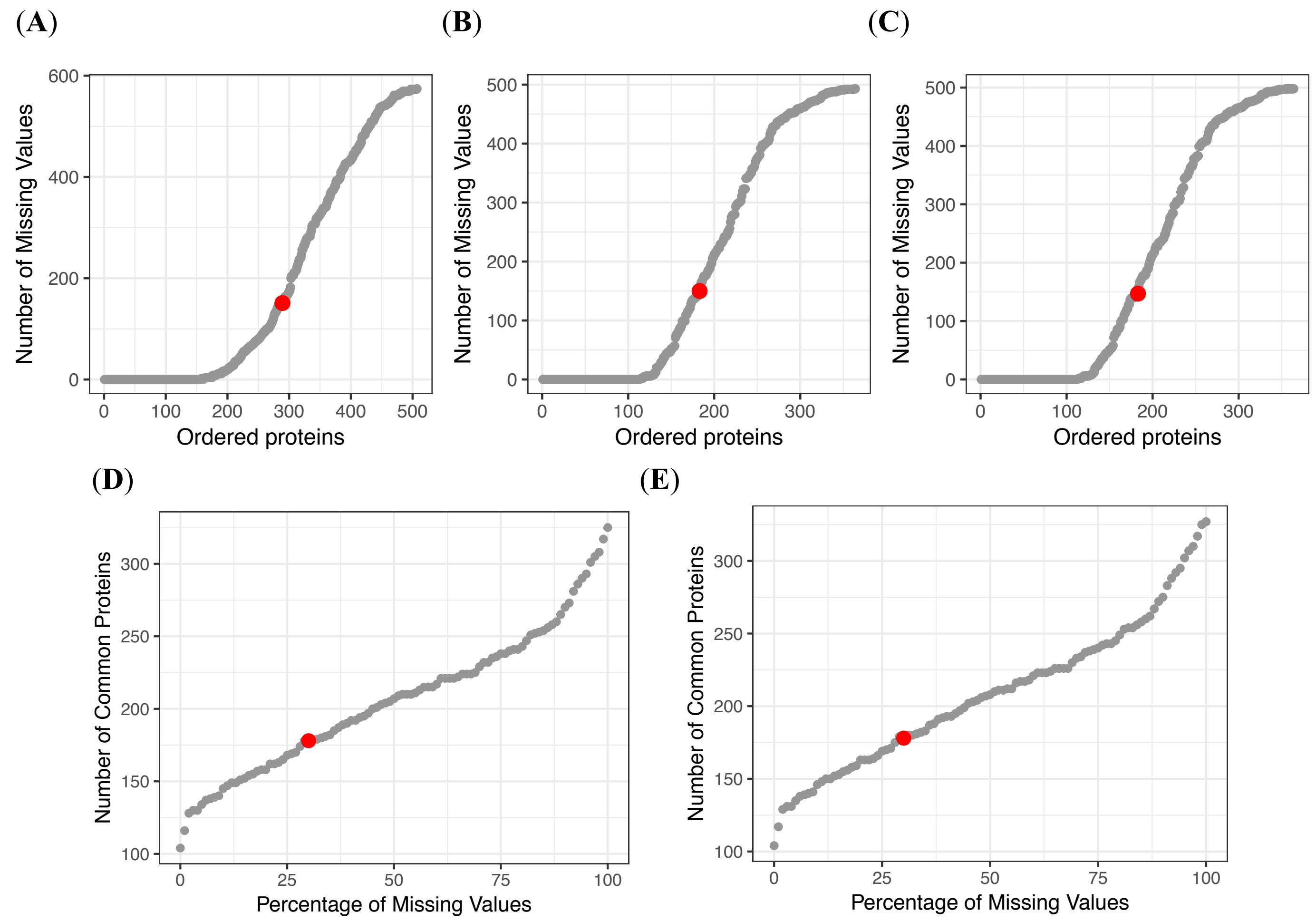

Figure S8. Data completeness. Number of missing values *versus* proteins – in increasing order regarding the number of missing values - for (**A**) C1, (**B**) C2A, and (**C**) C2B. The red dot indicates the 30% missing value threshold, below which proteins will be included. Number of common proteins between (**D**) C1 and C2A, and (**E**) C1 and C2B after applying a given missing value percentage threshold.

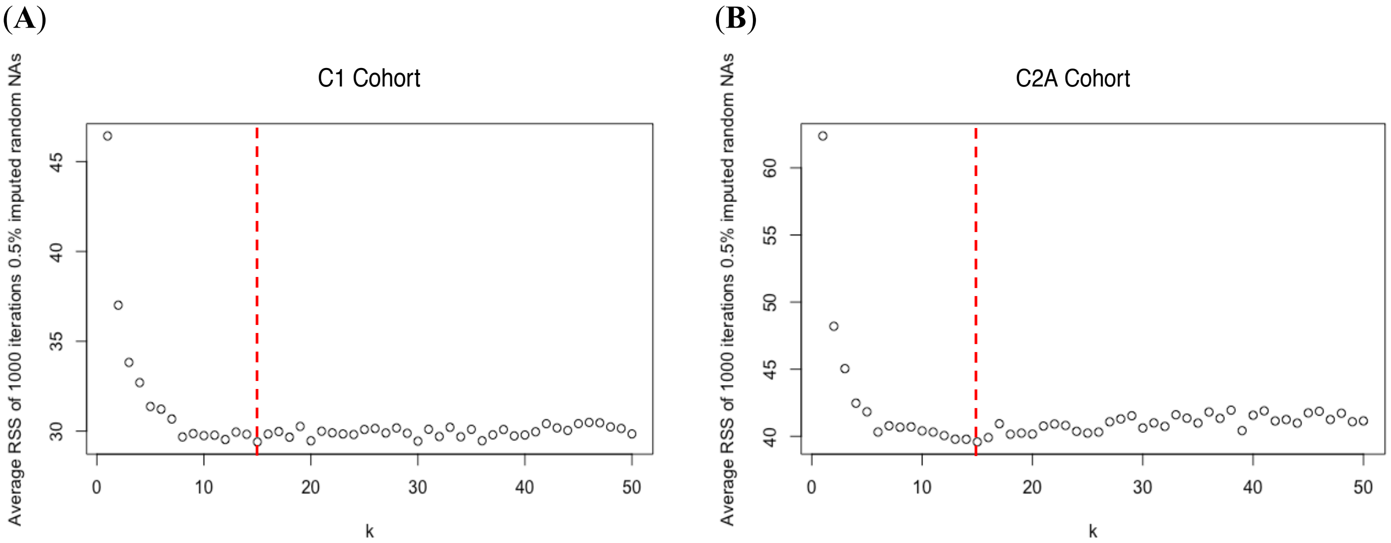

Figure S9. Determination of optimal k for KNN Imputation in both cohorts. 5% of data points are randomly removed at each iteration (1000 times) and the residual sum of squares (RSS) is computed.
